# The novel (TCTG)_n_ motif in *CNBP* expanded alleles: composition, dynamics and genotype–phenotype correlation in Myotonic dystrophy type 2 (DM2)

**DOI:** 10.1186/s40246-026-00954-7

**Published:** 2026-04-05

**Authors:** Federica Centofanti, Virginia Veronica Visconti, Maria Rosaria D’Apice, Marco Carlomagno, Simone Maestri, Dario Ciabini, Mario Bengala, Enrica Marchionni, Erica Frezza, Roberto Massa, Antonio Petrucci, Francesca Lupidi, Elena Pegoraro, Gabriele Siciliano, Matteo Garibaldi, Paola Origone, Massimo Delledonne, Marzia Rossato, Annalisa Botta, Giuseppe Novelli

**Affiliations:** 1 Department of Biomedicine and Prevention, Medical Genetics Section, University of Rome Tor Vergata, Rome, Italy; 2 Laboratory of Medical Genetics, Tor Vergata Hospital, Rome, Italy; 3 Department of Biotechnology, University of Verona, Verona, Italy; 4 Department of Systems Medicine (Neurology), University of Rome Tor Vergata, Rome, Italy; 5 Center for Neuromuscular and Neurological Rare Diseases, S. Camillo Forlanini Hospital, Rome, Italy; 6 Neurological Clinic, Azienda Ospedaliera Universitaria delle Marche, Ancona, Italy; 7 Department of Neuroscience, University of Padova, Padua, Italy; 8 Department of Clinical and Experimental Medicine, University of Pisa, Pisa, Italy; 9 Neuromuscular and Rare Disease Centre, Neurology Unit, Sant’Andrea Hospital, Rome, Italy; 10 Department of Neuroscience, Mental Health and Sensory Organs (NESMOS), SAPIENZA University of Rome, Rome, Italy; 11 Department of Neurosciences, Rehabilitation, Ophthalmology, Genetic and Maternal and Infantile Sciences, University of Genoa, Genova, Italy; 12 IRCCS Azienda Ospedaliera Metropolitana, Genoa, Italy

**Keywords:** Myotonic dystrophy type 2 (DM2), *CNBP* repeat expansions, QP-PCR, Variant repeats (VRs), Long read sequencing (LRS)

## Abstract

Introduction. Myotonic dystrophy type 2 (DM2) is an autosomal dominant disorder caused by (CCTG)_n_ repeat expansions in intron 1 of the *CNBP* gene. Recent evidence from long-read sequencing suggests these expansions may be more complex than previously recognized. Aim. To comprehensively characterize the composition, intergenerational dynamics, and clinical impact of novel (TCTG)_n_ motifs within the *CNBP* expanded alleles in a large DM2 cohort. Methods. We analyzed 100 genetically confirmed DM2 individuals (45 sporadic, 55 familial). The presence of (TCTG)_n_ blocks was detected using an optimized quadruplet-repeat primed PCR (QP-PCR) assay coupled with Sanger sequencing. In a subset of nine patients, Cas9-mediated enrichment followed by Nanopore Long-Read Sequencing (LRS) provided nucleotide-level resolution of the expanded alleles. Haplotype analysis was performed using STR markers. Results. We identified (TCTG)_n_ blocks at the 3′ end of the expansion in 88% of patients. This refined assay corrected nine initial false-negative diagnoses from standard testing. LRS analysis confirmed the composition and revealed the dynamics of the (TCTG)_n_ tract in familial transmission, showing a tendency for contraction and, in one case, complete loss. Genotype–phenotype correlation analysis indicated that the presence of the (TCTG)_n_ motif acts as a disease modifier, significantly influencing the age of onset. Conclusion. The detailed characterization of the *CNBP* expansion reveals the novel (TCTG)_n_ component that is integral to the DM2 genotype. Understanding its composition and dynamics enhances diagnostic accuracy and provides a new framework for genetic counselling, prognostic stratification and future personalized therapies. .

**Supplementary Information:**

The online version contains supplementary material available at 10.1186/s40246-026-00954-7.

## Introduction

Myotonic dystrophy type 2 (DM2; MIM#602668), also known as proximal myotonic myopathy (PROMM), is an autosomal dominant, slowly progressive multisystem disorder [1, 2]. DM2 primarily affects proximal muscles (hips and shoulders) with symptoms including muscle pain (myalgia), cramps, myotonia (impaired muscle relaxation), muscle weakness and wasting (dystrophic changes), cataracts, and potential involvement of other organ systems, such as cardiac and endocrine abnormalities [3, 4]. The genetic basis of DM2 is a (CCTG)_n_ tetranucleotide repeat expansion in intron 1 of the *CNBP* gene (MIM*116955) on chromosome 3q21.3. In healthy individuals, the *CNBP* repeat locus comprises a complex (TG)_v_(TCTG)_w_(CCTG)_x_ motif, typically interrupted by one or more NCTG motifs (GCTG, TCTG, or ACTG), which contribute to repeat stability [5–7]. This can be represented as (TG)_v_(TCTG)_w_(CCTG)_x_(NCTG)_y_(CCTG)_z_ [6, 8]. It is necessary to note that, accordingly to the HGVS nomenclature the correct *CNBP*-DM2 motif description at the genomic level should be GCAG[n]ACAG[o]AC[p]) [9], nevertheless in this paper the conventionally used nomenclature has been adopted to be more comparable with previous sequencing studies on the genetics of the DM2 locus. Stable normal alleles contain fewer than 25 CCTG repeats, while pathogenic expansions range from approximately 75 to over 11,000 repeats, among the largest reported in repeat expansion disorders. Alleles with 27–74 CCTG repeats (the “grey area”) are unstable in the germline and somatic cells with unknown clinical significance and require sequence analysis to better estimate the exact composition of the (CCTG)_n_ repeat since the lack of interruptions within the (CCTG) array may lead to unstable premutated alleles [10]. Unlike Myotonic dystrophy type 1 (DM1; MIM #160900), where the (CTG)_n_ repeat length in the *DMPK* gene roughly correlates with disease severity, no genotype–phenotype correlation has been established in DM2 patients, despite the wide range of expansion sizes [6, 11–13]. The DM2 mutation exhibits marked somatic instability, with an increasing length over time within individuals. Still, intergenerational expansion (genetic anticipation) is rare, unless genetic modifiers intervene to worsen the clinical symptoms [14–16], and congenital DM2 has not been reported so far. Nevertheless, the uncertainty regarding clinical anticipation and parent-of-origin effects in DM2 requires further investigation. The primary DM2 pathogenic mechanism involves RNA gain-of-function toxicity, leading to the formation of CCUG-containing ribonuclear foci and spliceopathy, primarily through sequestration of splicing regulators involved in the early phases of transcript processing [17]. Additional findings suggest that DM2 molecular pathogenesis is more complex, involving changes in gene expression and translation efficiency, non-ATG translation (RAN translation), microRNAs (miRNAs) and circular RNAs (circRNAs) deregulation [16]. Molecular diagnosis of DM2 presents challenges due to the large size, high GC content, and somatic mosaicism of the (CCTG)_n_ expansions. According to international guidelines, current best practice for DM2 genetic testing relies mainly on PCR-based approaches [12, 18]. Standard diagnostic workflows begin with short-range PCR (SR-PCR) to identify normal-sized alleles. If only one allele is detected, indicating a potential expansion in the other allele, further testing is performed using bidirectional quadruplet-repeat primed PCR (QP-PCR) and blotting of long-range PCR (LR-PCR), achieving an approximately 99% detection rate. However, these methods do not precisely determine the length of large expansions, for which Southern blotting of digested genomic DNA is required, albeit with a sensitivity of only ~ 80%. Southern blotting is also a time-consuming method, requires substantial DNA, and is not routinely used in many diagnostic laboratories. Moreover, none of these methods provides single-nucleotide resolution or accurately assesses minor alleles and somatic mosaicism. Next-generation sequencing (NGS) methods, especially short-read whole-genome sequencing (WGS), have recently become important diagnostic tools for tandem repeat (TR) disorders, including DM2, enabling more accurate characterisation of repeat motifs. Lojova et al. showed that short-read WGS can describe normal *CNBP*-complex motif alleles and detect premutations and expansions [9]. While these short-read technologies are increasingly effective for genotyping and identifying the presence of expanded motifs, they often struggle to fully resolve the complete architecture of very large or complex expansions [19]. Although second-generation (short-read) sequencing can detect expanded alleles in DM2 [9], it does not produce reads long enough to span the entire repeat region, precluding full characterisation of repeat structure and composition [20]. Third-generation (long-read) sequencing technologies, such as Oxford Nanopore Technologies (ONT) and PacBio Single Molecule Real Time (SMRT) sequencing, offer the potential to overcome these limitations by analysing DNA fragments of several kilobases, including large repetitive elements [19]. Long-read sequencing (LRS) methods are based on single-molecule sequencing, thus avoiding clonal amplification of sequencing-by-synthesis platforms, and can handle high GC content, especially through PCR-free protocols. In our previous work, we evaluated a combination of CRISPR/Cas9-based enrichment (Cas9-enrichment) and ONT sequencing for analysing *CNBP* expansions. Using this approach, we sequenced full-length (CCTG)_n_ expansions in nine DM2 patients, including one allele of 47 kb. We detected a previously unreported (TCTG)_n_ motif at the 3′ end of the *CNBP* expansion in seven of these patients [21]. In this paper, we aim to extend our pilot study to assess the frequency and the genotype–phenotype correlations of *CNBP* expanded alleles containing the (TCTG)_n_ motif in a larger cohort of DM2-positive patients using an optimized QP-PCR method, which is designed to amplify selectively TCTG-containing expanded alleles [21]. Moreover, the *CNBP* locus has been fully characterised using ONT-LRS in a sub-cohort of selected DM2 patients (*n* = 9) to study the composition and intergenerational dynamics of the DM2 “pure” and TCTG-containing expanded alleles (henceforth defined as (CCTG)_n_+(TCTG)_n_ in the text).

## Results

### *CNBP* expanded alleles containing (TCTG)_n_ motifs 3′ to the (CCTG)_n_ repeated array are the most common mutations in DM2 patients

To establish the frequency of the novel (TCTG)_n_ motif in a larger cohort of DM2 patients, we used an optimised version of the QP-PCR method previously described in Alfano et al. [21]. as reported in the Materials and Methods section. To this purpose, we re-analysed 100 genetically confirmed DM2 patients (previously characterized using a combination of PCR-based techniques, including SR-PCR, LR-PCR and bidirectional QP-PCR), consisting of 41 males and 59 females, with a mean age of 53.9 years (± 34 years). Fifty-five patients were classified as familial cases, while the remaining 45 were classified as single cases with no other affected family members available for the analysis (Table 1).

**Table 1:** Frequency of the (TCTG)_n_ motifs within *CNBP* expanded alleles in our cohort of DM2 patients

DM2 patients	Total number (*n* = 100)	Presence of (TCTG)_*n*_ motif	
		Yes	No
Male	41	39	2
Female	59	49	10
Total	100	88	12
		yes	no
Familial	55	53	2
Single Case*	45	35	10
Total	100	88	12

*referred to patients with no other family members available for the analysis

**Fig. 1:**
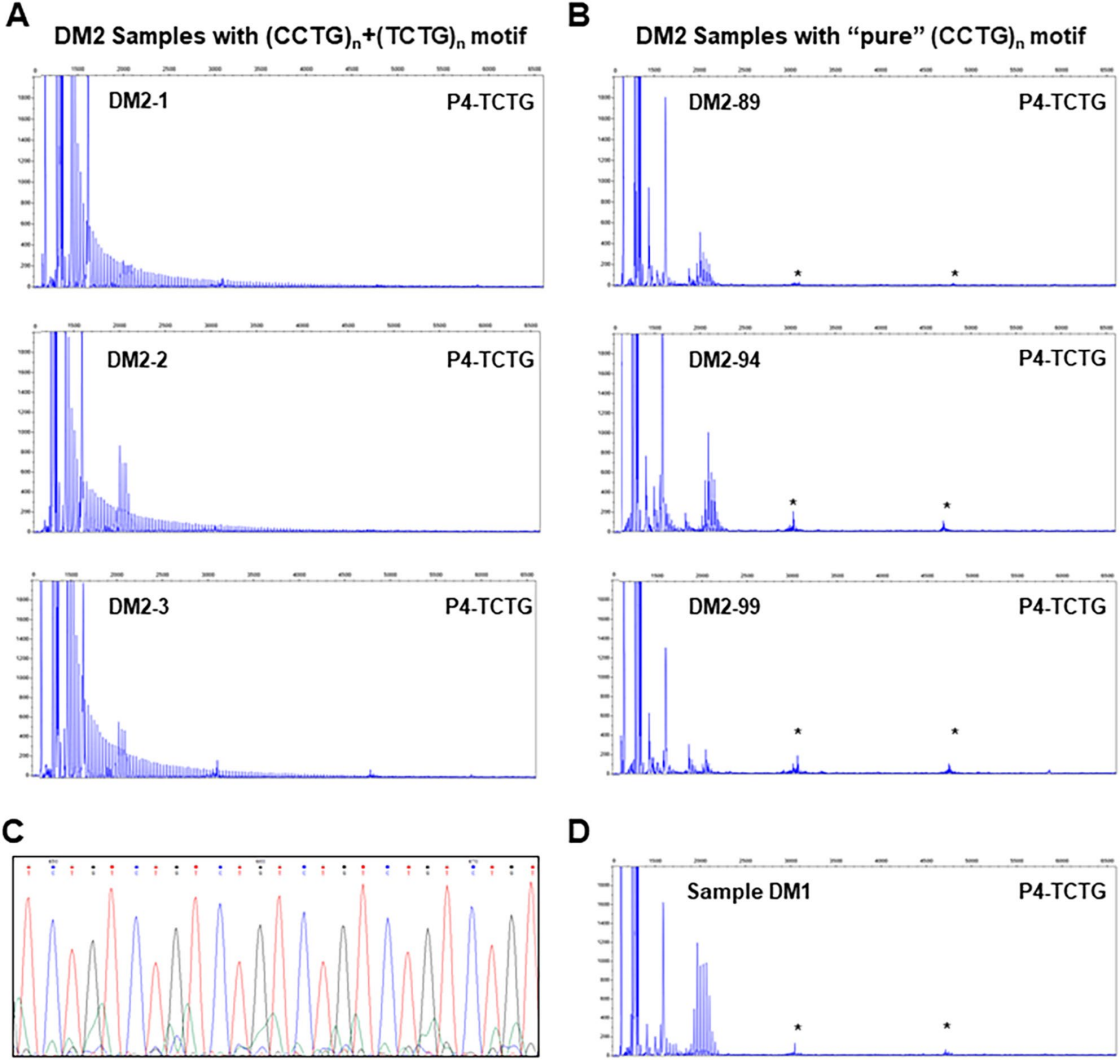
Analysis of the (TCTG)_n_ motif by quadruplet-repeat primed PCR (QP-PCR) and Sanger sequencing. **A** Representative QP-PCR profiles of three DM2 patients (DM2-1, DM2-2, and DM2-3) carrying the (TCTG)_n_ block at the 3′ end of the (CCTG)_n_ array. In these samples, the presence of the (TCTG)_n_ motif is clearly indicated by a characteristic continuous ladder of peaks out of the normal range, resulting from the specific binding of the P4-TCTG primer to the expanded block. **B** Representative QP-PCR profiles of three DM2 patients (DM2-89, DM2-94, and DM2-99) without the (TCTG)_n_ block. These profiles lack the ladder signal typical of expanded alleles. **C** Representative Sanger sequencing of the QP-PCR products confirms the presence of the TCTG sequence at the 3′ end of the DM2 expansion. **D** QP-PCR profile of a DM1 sample, used here as a negative control. This profile illustrates the typical signals of non-expanded alleles; notably, the pattern in D resembles the profiles of the three patients in B, confirming the absence of the (TCTG)_n_ block in these individuals. Asterisks (*) indicate nonspecific signals, also visible in the negative control (Panel D) and previously described in Alfano et al., 2022 [21]

Our findings revealed that 88/100 DM2 patients (88%) exhibited (TCTG)_n_ blocks at the 3′ end of the (CCTG)_n_ array (Fig. 1A), whereas only 12/100 (12%) patients displayed a continuous (CCTG)_n_ expansion pattern (Fig. 1B). The (TCTG)_n_ motif was present in about 4% of familial cases and 29% of single cases.

**Fig. 2:**
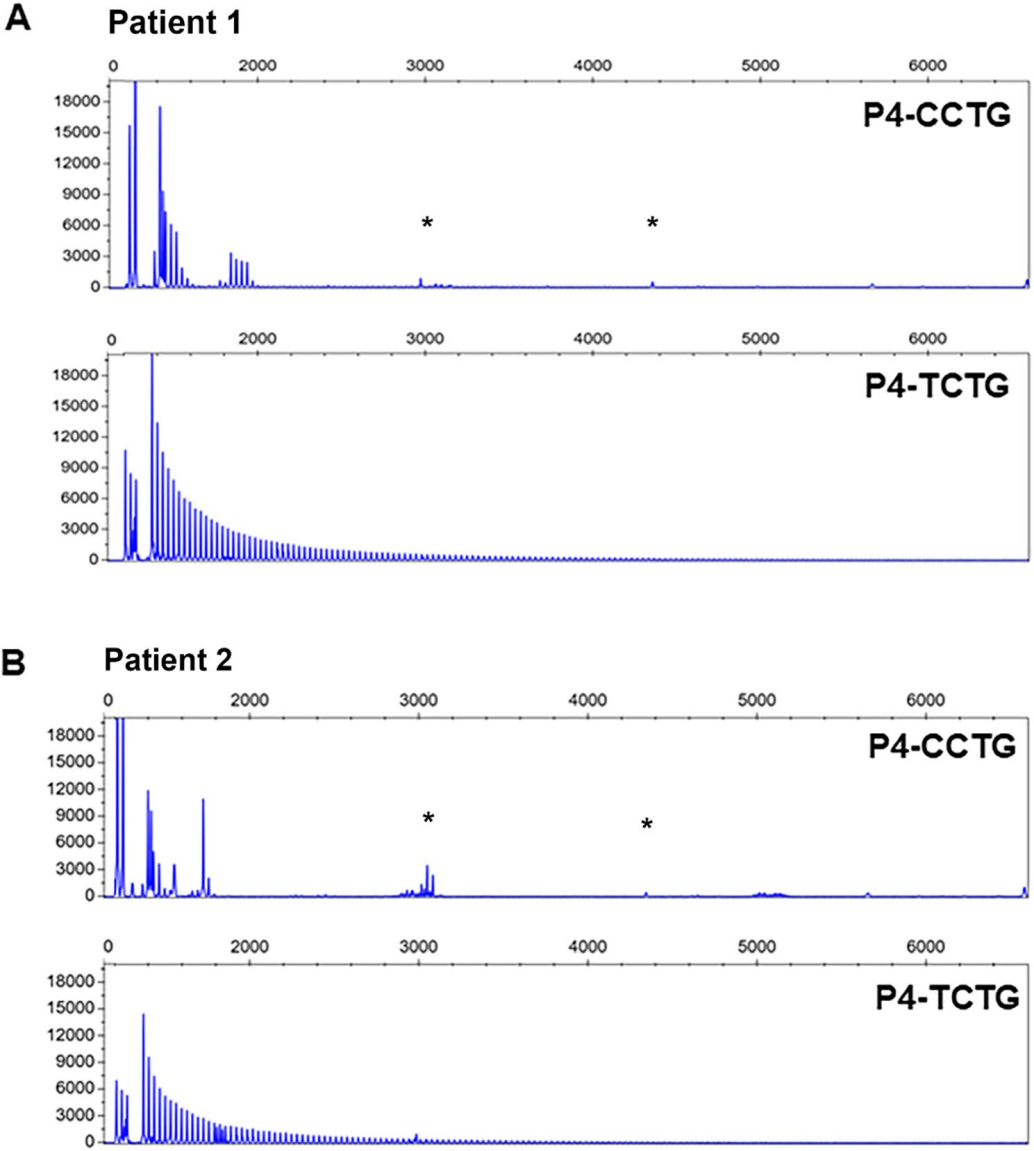
QP-PCR analysis of two representative DM2 patients showing apparently negative results using standard QP-PCR performed at the 3′ end of the expanded array. **A** Patient 1: Upper panel shows negative results using the P4-CCTG primer; Lower panel shows the characteristic ladder profile of expanded alleles obtained using the P4-TCTG primer. **B** Patient 2: Upper panel shows negative results using the P4-CCTG primer; Lower panel shows the characteristic ladder profile of expanded alleles obtained using the P4-TCTG primer. Asterisks (*) indicate nonspecific signals, and previously described in Alfano et al., 2022 [21]

The presence of the (TCTG)_n_ motif was further confirmed through Sanger sequencing of the QP-PCR products in all 88 DM2 patients (Fig. 1C), providing robust support for our findings (Table 1). The above-described protocol was also used to re-evaluate 9 complex DM2 cases, who tested negative with the standard QP-PCR test at the 3′end of the (CCTG)_n_ repeated motif, whereas both the 5′-QP-PCR and LR-PCR were demonstrating the presence of a *CNBP* expanded allele. To verify whether the failure of the standard 3′-QP-PCR was due to the presence of the (TCTG)_n_ block in the 3′ end of the (CCTG)_n_ array, we use our optimized QP-PCR with the P4-TCTG primer. This analysis revealed an electrophoretic profile compatible with the presence of (TCTG)_n_ downstream of the CCTG expansion in these DM2 samples (Fig. 2), which likely did not allow the binding of the P4-(CCTG)_5_ primer, thus explaining the failure of the standard 3′ QP-PCR assay.

### Genotype–phenotype correlation in DM2 patients carrying (CCTG)_n_+(TCTG)_n_ and (CCTG)_n_ “pure” repetitions

**Fig. 3:**
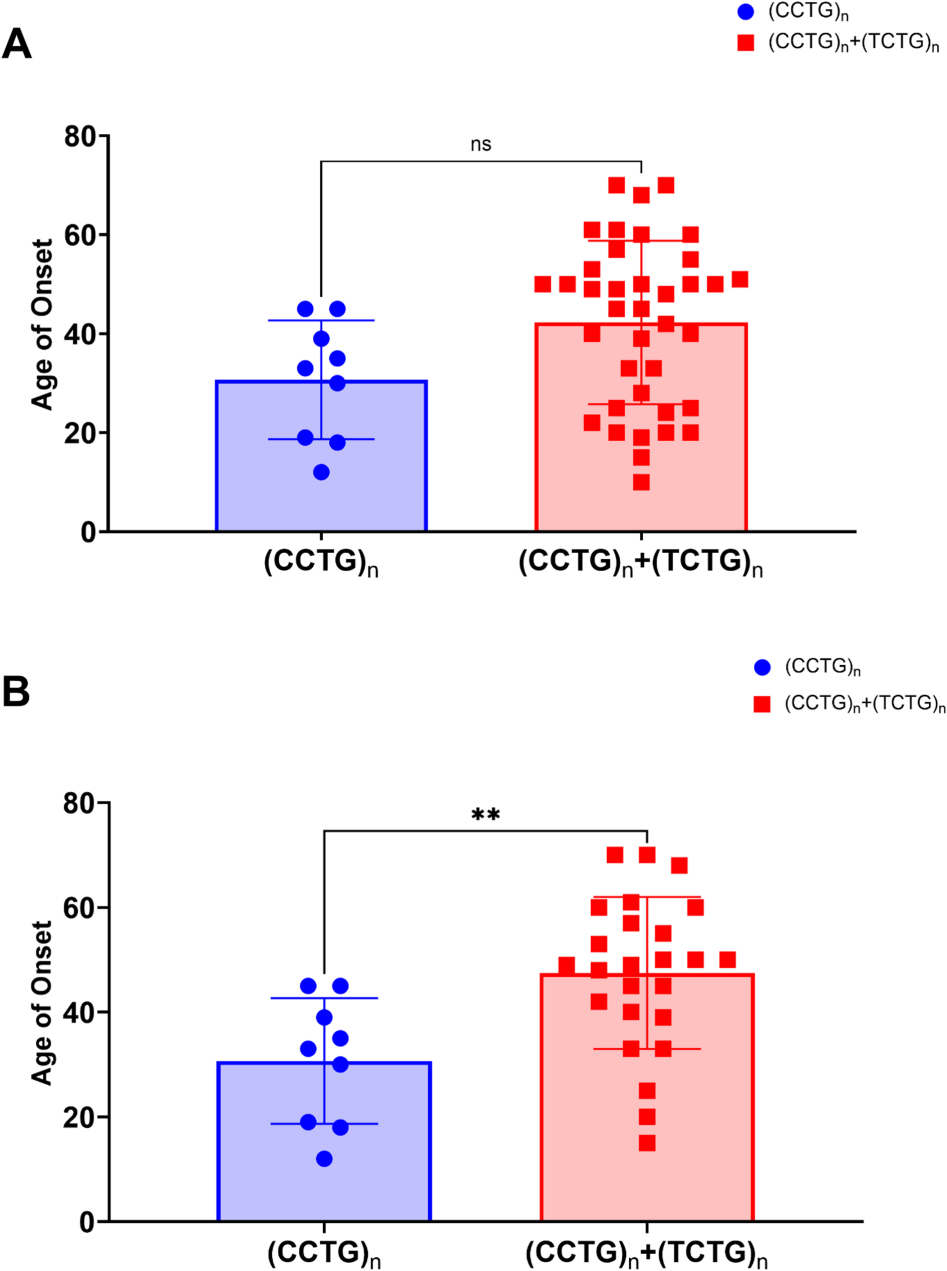
Comparison of disease age of onset between DM2 patients with “pure” (CCTG)_n_ and (CCTG)_n_+(TCTG)_n_ *CNBP* expanded alleles. (A) Age of onset in the total cohort of DM2 patients (*n* = 56), including both familial and sporadic cases, categorised by the presence of “pure” (CCTG)_n_ (blue box; *n* = 10) or (CCTG)_n_+(TCTG)_n_ alleles (red box; *n* = 46). (B) Age of onset of DM2index cases (*n* = 32), categorised by the presence of “pure” (CCTG)_n_ (blue box; *n* = 9) or (CCTG)_n_+(TCTG)_n_ alleles (red box; *n* = 23). Results are represented as the mean ± SD (ns = not significant; ** *p* < 0.01; by Unpaired T-test)

Given the high frequency of the (TCTG)_n_ motif and the lack of data regarding the clinical phenotype associated with these newly described *CNBP* expanded alleles, we decided to re-evaluate the clinical symptoms in a selected sub-cohort of 56 DM2 patients for which haemato-chemical and clinical evaluations, including instrumental tests, were available. The following clinical symptoms were considered: myotonia, myalgia, cataract, asthenia, hyperCKaemia (presence of elevated serum creatine kinase (CK) levels), cramps, muscle weakness, heart disease, diabetes and hypothyroidism. Our cohort consisted of 10 patients which did not show the presence of the (TCTG)_n_ motif 3′ to the (CCTG)_n_ array (hereafter referred to as “pure’**”** (CCTG)_n_ motif) (2 males and 8 females), with a mean age of 45.25 years (± 13.29 years) and 46 patients carrying the (TCTG)_n_ motif (20 males and 26 females), with a mean age of 55.18 years (± 17.49 years). We observed that the “pure” (CCTG)_n_ motif is more frequent in females (77.8%) compared to male DM2 patients (22.2%) and is associated with a lower mean age of disease onset compared to DM2 patients with the (TCTG)_n_ motif (30.7 years ± 12 years vs. 42.6 years ± 16.51 years), with a *p*-value very close to significance (*p* = 0.0534) (Fig. 3).

**Fig. 4:**
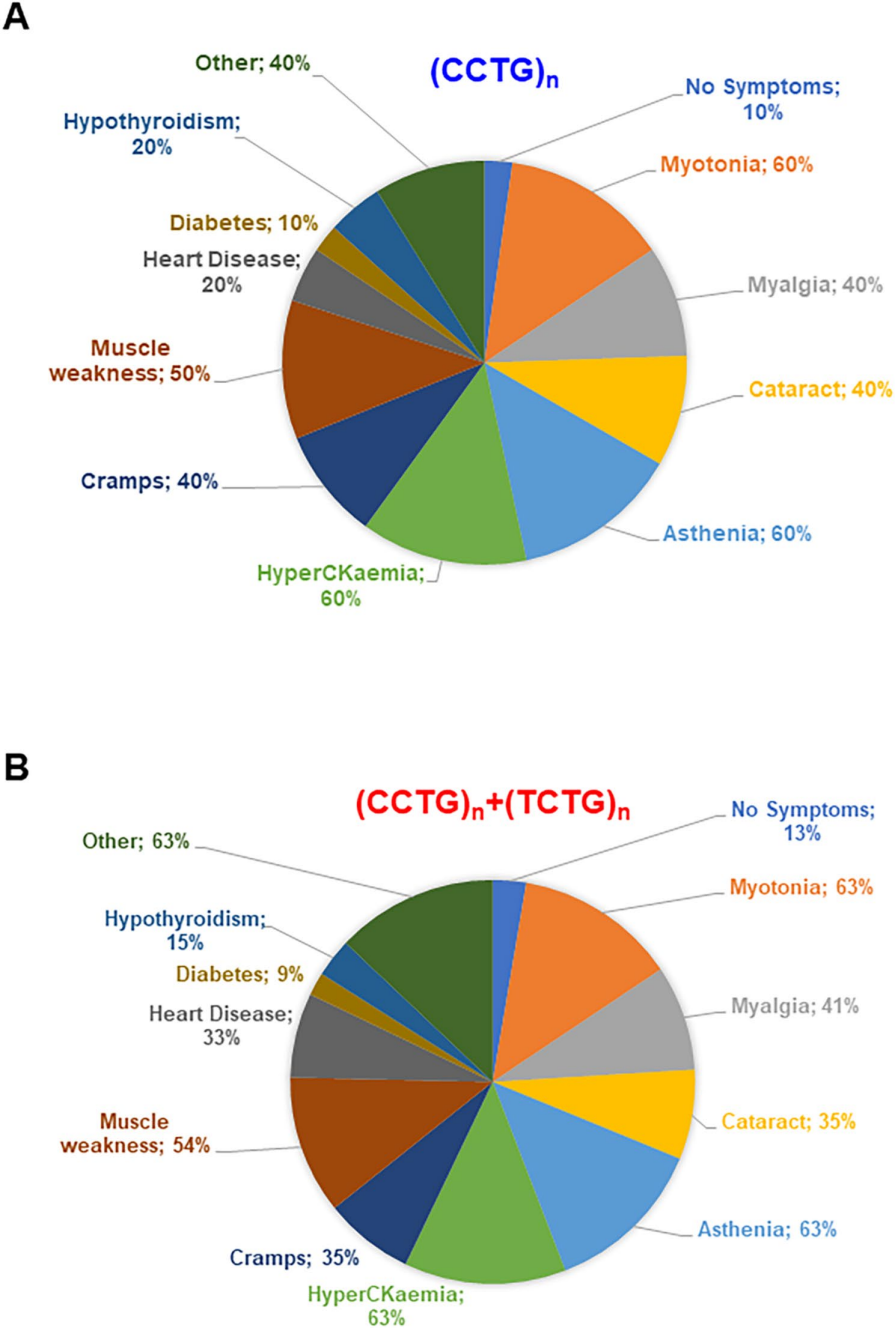
Main clinical symptoms of DM2 with “pure” (CCTG)_n_ (**A**) and (CCTG)_n_+(TCTG)_n_ (**B**) *CNBP* expanded alleles

To avoid a potential bias in this analysis due to the early detection of the clinical symptoms in familial cases, we decided to repeat the same correlation study, including only symptomatic index cases (9 vs. 23 patients). Interestingly, we found that the presence of the “pure” (CCTG)_n_ motif is significantly associated with a decrease in the age of disease onset (** *p* = 0.0039). However, given the limited number of DM2 patients without the (TCTG)_n_ repetitions, further studies in larger cohorts of DM2 patients are needed to confirm these preliminary results. We were also able to determine the length of the repeated tract in 6 DM2 patients with the “pure” (CCTG)_n_ motif, by conventional Southern blot analysis on digested genomic DNA. This analysis showed that the *CNBP* expanded alleles ranged from 6.5 to 9.4 kb to about 40 kb (Supplement Fig. 1). In agreement with published data, we did not observe a significant correlation between age at onset and the maximum DM2 mutation length of *CNBP* alleles containing a “pure” (CCTG)_n_ motif (*r* = 0.3339; *p* = 0.5333). This finding suggests that nucleotide composition, rather than *CNBP* expansion length, may play a role in determining disease outcome (Supplementary Tables 1 and Supplementary Fig. 2). We must, however, consider that age-dependent mosaicism could confound this analysis, given the variability in the ages of the patients analyzed. We therefore wanted to evaluate whether it was possible to stratify “pure” (CCTG)_n_ compared to (CCTG)_n_+(TCTG)_n_ DM2 patients based on the clinical phenotype. In patients carrying “pure” *CNBP* expansion, the clinical symptoms were as follows: 60% (6/10) experienced myotonia, 60% (6/10) presented with hyperCKaemia, 60% (6/10) suffered from asthenia, 50% (5/10) showed muscle weakness, 40% (4/10) reported myalgia, 40% (4/10) had cataracts, 40% (4/10) dealt with cramps, 20% (2/10) had hypothyroidism, 20% (2/10) had heart disease, 10% (1/10) were diabetic, 10% (1/10) had no symptoms, and 40% (4/10) exhibited other symptoms (Fig. 4A).

In DM2 patients carrying (TCTG)_n_ repetitions, the spectrum of symptoms was as follows: 63% (29/46) had myotonia, 63% (29/46) experienced asthenia, 63% (29/46) exhibited hyperCKaemia, 54.4% (25/46) showed muscle weakness, 41.3% (19/46) reported myalgia, 34.8% (16/46) presented with cataracts, 34.8% (16/46) dealt with cramps, 32.6% (15/46) had heart disease, 15.2% (7/46) had hypothyroidism, 13% (6/46) showed no symptoms, 8.7% (4/46) were diabetic, and 63% (29/46) reported other symptoms (Fig. 4B). No notable differences in clinical symptoms were observed between the two groups, suggesting that the mere presence or absence of the (TCTG)_n_ motif is insufficient to stratify the clinical phenotype by severity.

### Molecular characterisation of the DM2 mutation through Cas9-mediated sequencing to study the length, composition, and intergenerational dynamics of *CNBP* expanded alleles

To further validate our QP-PCR results, nine DM2 patients have been further analyzed by LRS using an optimized protocol of Cas9-mediated enrichment coupled with the most recent ONT sequencing (R10.4.1) as described in the Materials and Methods section. Among them, 3 were single cases and 6 patients belonged to two DM2 families (A and B, Table 2).

**Table 2:**
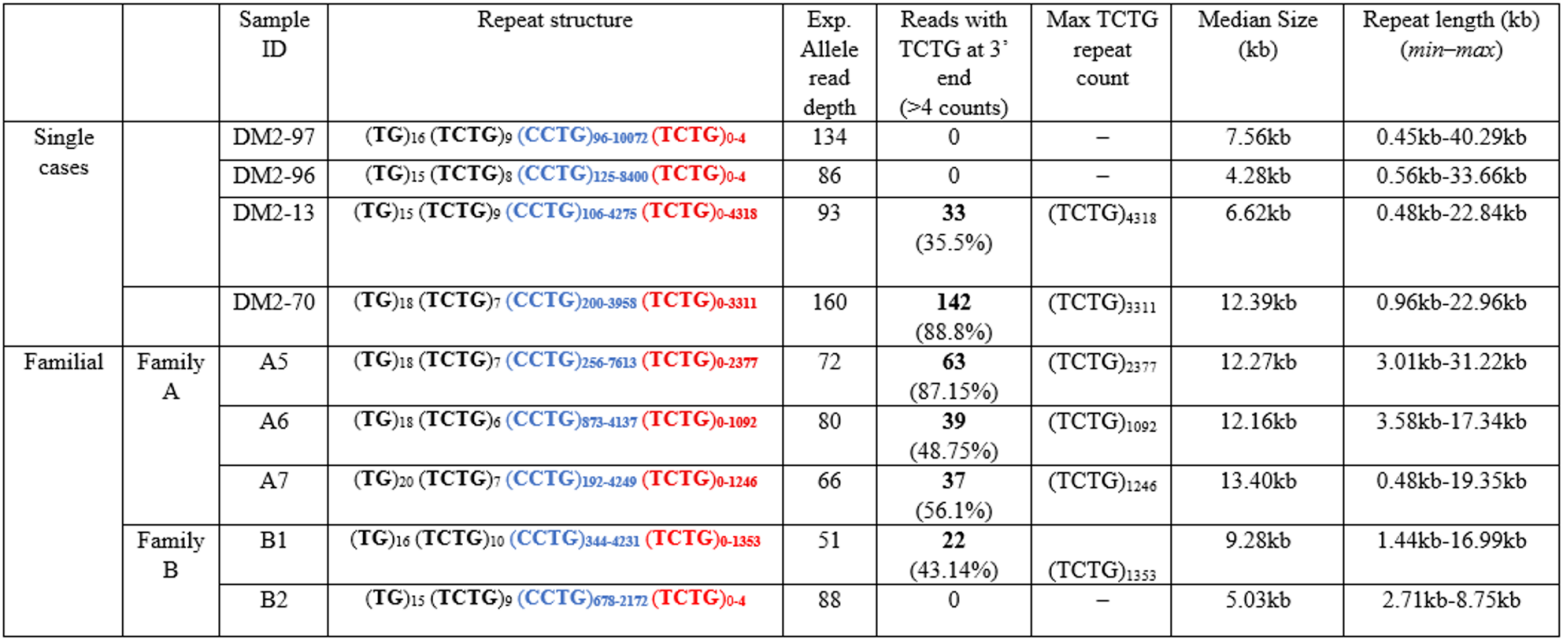
*CNBP* repeat analysis based on Cas9-mediated sequencing of the expanded alleles

For each patient, the table shows the characteristics of expanded *CNBP* alleles based on the analysis of ONT sequencing data, in terms of length and structure. The expanded (CCTG)_x_ are coloured in blue and (TCTG)_y_ in red. The table also indicates the fraction of sequencing reads from the expanded alleles carrying the (TCTG)_n_ motif at the 3′ end of the *CNBP* microsatellite expansion

Based on QP-PCR analysis, the (CCTG)_n_+(TCTG)_n_ was present in 6/9 DM2 patients, whereas 3/9 affected individuals did not show the presence of the (TCTG)_n_ motif (Table 2). ONT sequencing reads were mapped to human reference genome (hg38) and, for all patients, we detected a considerable amount of reads fully spanning the *CNBP* expanded alleles, achieving a median coverage of 555x across the region of interest. The maximal expansion length per patient ranged from 8.75 kb to 40.29 kb, corresponding to a median number of repetitive quadruplets from 2,187 to 10,072 units (Table 2). This analysis also demonstrated a pronounced intra-patient mosaicism as observed in our previous work [21]. Consistent with QP-PCR data, the expanded (TCTG)_n_ motif was identified in all members of Family A and in one member of Family B (B1), while it was not detected in patient B2, who was confirmed to carry “pure” (CCTG)_n_ expanded alleles. In most cases, the fraction of reads from the expanded allele carrying the (TCTG)_n_ motif was a large amount (median 56%), reaching up to 88.8% in patient DM2-70 (Table 2), while the length of this motif was always shorter than that of the (CCTG)_n_ motif. As observed in our previous cohort [21], the (TCTG)_n_ array also showed a very variable size, both inter- and intra-patient (Table 2). Overall, the expansion size and structural configuration determined by ONT were highly concordant with those obtained by QP-PCR. A consistent fraction of sequencing reads did not display either the (CCTG)_n_ or the (TCTG)_n_ motif, as indicated by the presence of grey segments within individual reads, representing regions including not-classified repeat units. As shown more in detail in Supplementary Fig. 3 these regions may include higher-rate of ONT sequencing errors or additional repeat motifs that would, however, require further analysis.

**Fig. 5:**
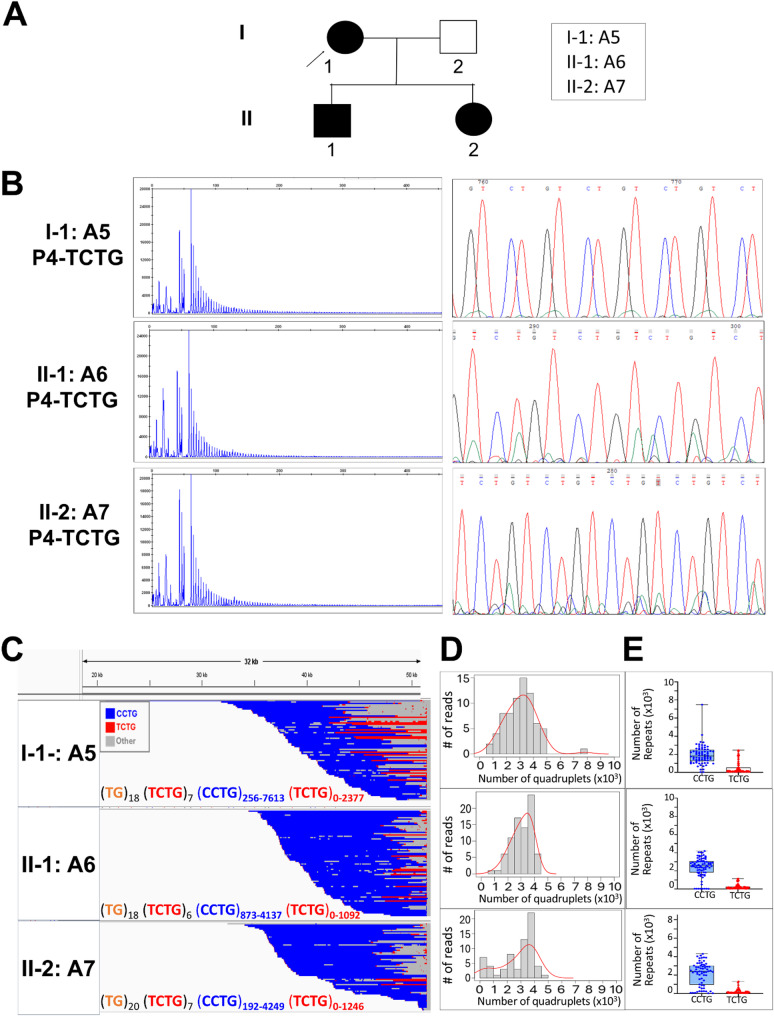
Analysis of the (TCTG)_n_ motif DM2 Family A. **A** Genetic pedigree showing the relationship among familial cases analysed in this study. The age at sampling for each family member was as follows: I-1: A5, 54 years; II-1: A6, 29 years; II-2: A7, 25 years. The rows indicate the index cases analysed. **B** QP-PCR profiles, Sanger sequencing, and **C** Integrative Genomics Viewer (IGV) visualization (32 kb windows) of ONT-targeted sequencing data that show the presence of the (TCTG)_n_ block in all 4 members at the 3′ ends of the (CCTG)_n_ expansions. Complete reads were aligned at the 3′ end in order to identify the repeat pattern that characterizes the expanded microsatellite locus. Each motif in the expanded alleles was visualized using a different colour, as indicated in the key. **D** Abundance of quadruplets identified in each patient. The y-axis shows the number of ONT reads with a certain number of repeats, whereas the x-axis shows the number of quadruplet repeats identified. ONT reads were grouped into 500 bp bins. The red line represents the estimated kernel density of the underlying solid gray distribution of ONT reads. **E** Size distribution of (CCTG)_n_ and (TCTG)_n_ motifs identified within each individual read. Boxes represent the interquartile range (IQR) of lengths, the horizontal line is the median, whiskers and outliers are plotted according to Tukey’s method. Unclassified motif runs were not considered

**Fig. 6:**
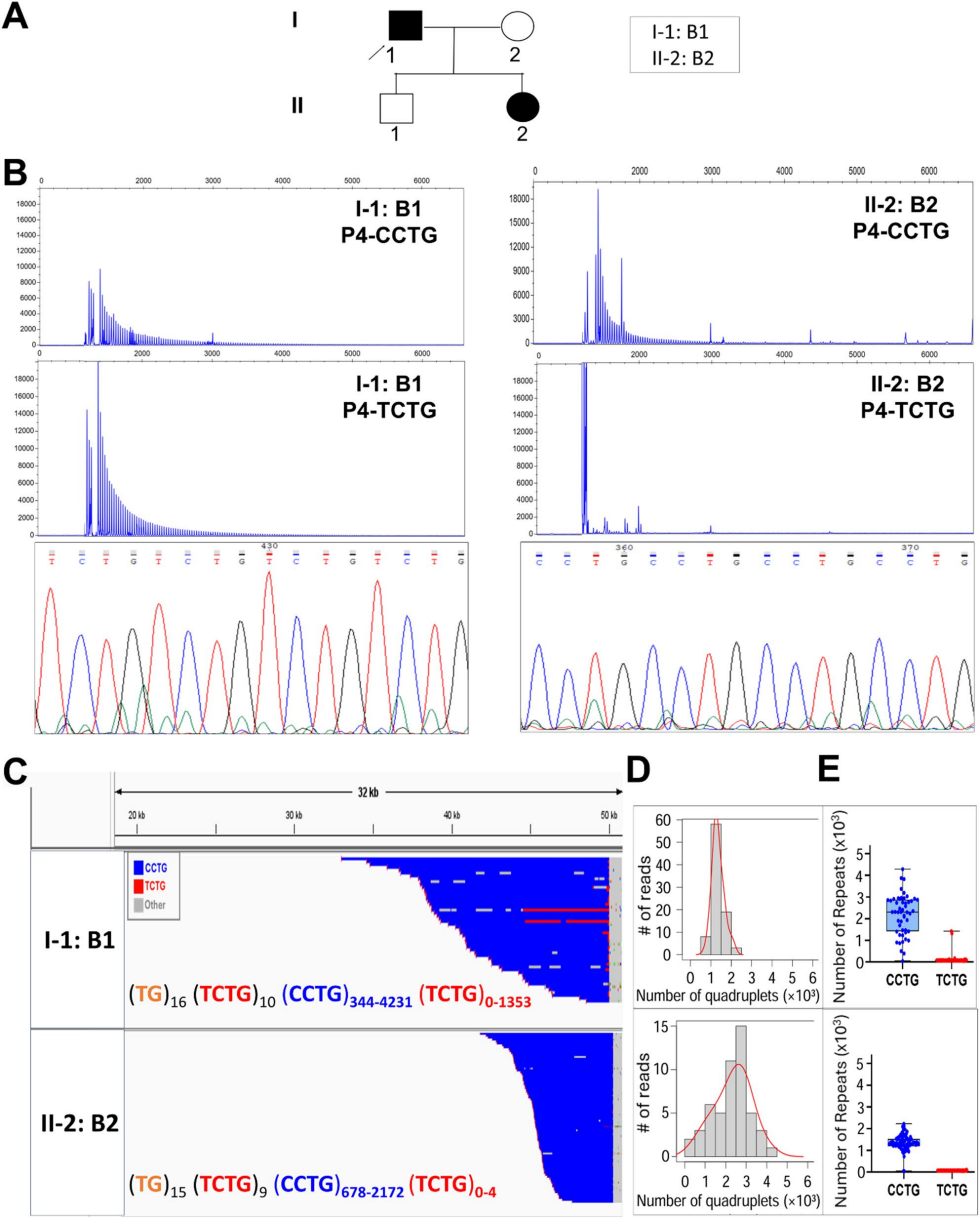
Analysis of the (TCTG)_n_ motif in DM2 Family B. **A** Genetic pedigree showing the relationship among familial cases analysed in this study. The age at sampling for each family member was as follows: I-1: B1, 76 years; II-2: B2, 42 years. The rows indicate the index cases analysed. **B** QP-PCR profiles, Sanger sequencing, and **C** Integrative Genomics Viewer (IGV) visualization (*32*kb windows) of ONT-targeted sequencing data. Complete reads were aligned at the 3′ end *to* identify the repeat pattern that characterizes the expanded microsatellite locus. Each motif in the expanded alleles was visualized using a different colour, as indicated in the key. Samples *B2* contain*s* a ‘pure’ (CCTG)_n_ expansion (blue) whereas samples *B1* contains the unexpected (TCTG)_n_ motif (red) downstream of CCTG. **D** Abundance of quadruplets identified in each patient. The y-axis shows the number of ONT reads with a certain number of repeats, whereas the x-axis shows the number of quadruplet repeats identified. ONT reads were grouped into 500 bp bins. The red line represents the estimated kernel density of the underlying solid gray distribution of ONT reads. **E** Size distribution of (CCTG)_n_ and (TCTG)_n_ motifs identified within each individual read. Boxes represent the interquartile range (IQR) of lengths, the horizontal line is the median, whiskers and outliers are plotted according to Tukey’s method. Unclassified motif runs were not considered

To study the intergenerational dynamics of the (TCTG)_n_-containing *CNBP* expanded alleles, we analysed, by QP-PCR, 16 DM2 families (a total of 23 meiotic transmissions) from our cohort. Only 6/23 individuals (26.1%) inherited the *CNBP* paternal expanded allele, whereas the mutation was maternally inherited in 17/23 patients (73.9%). Interestingly, the (TCTG)_n_ motif was consistently inherited, with the exception of two DM2 families in which the repeat was lost across meiotic transmissions. LRS analysis was therefore used to deeply study the dynamics of the DM2 mutation in Family A and B. This analysis is essential because it allows to study, for the first time, the intergenerational transmission of (TCTG)_n_-containing expanded alleles in terms of motif configuration and repeat composition. Family A is composed of mother (A5) and her two sons (A6 and A7) (Fig. 5); Family B is composed of father (B1) and his daughter (B2) (Fig. 6).

Intergenerational transmission of the DM2 mutation within Family A confirmed the conservation of the (CCTG)_n_+(TCTG)_n_ motif, with different configurations among all affected family members. LRS analysis of the structure and composition of the expanded *CNBP* allele in this family reveals a variation in repeat sizes when considering the maximum repeated length: an apparent maternal contraction of the DM2 mutation from A5 to her two children, A6 and A7. Specifically, there is a reduction in the maximum number of both types of repetitions: the (CCTG)_n_ motif decreases from about 7500 repeats to approximately 4000 units, and the (TCTG)_n_ motif drops from around 2400 repeats to about 1000 in both children. Despite these changes, the median of the expanded *CNBP* among the three components remains nearly unchanged (Fig. 5; Table 2). The fraction of reads from expanded allele carrying the (TCTG)_n_ motif was 87.15% in the mother (A5), while in the children (A6 and A7) were about 50%. From the clinical point of view, the mother A5 (age 54 years and age at onset 50 years) presents typical DM clinical symptoms, whereas her two sons did not show any symptoms at the age of examination (ages 29 and 25). Interestingly, in Family B, we observed for the first time the loss of the (TCTG)_n_ tract, leading to a more consistent contraction of the *CNBP* expanded alleles in the patient B2 through paternal transmission (minimum, median and maximum repeat length). Analysing the structure and composition of the expanded *CNBP* allele in this family, we not only observed the loss of (TCTG)_n_ in the daughter B2 but also an apparent contraction of the (CCTG)_n_ motif that decreases from about 4200 to about 2100 units (Fig. 6, Supplementary Fig. 4 and Table 2). Clinical evaluation of both family members showed multisystemic clinical symptoms with an earlier disease age of onset in B2 (50 vs. 30 years), accordingly to our previous observation.

### Haplotype analysis of the *CNBP* expanded region containing (CCTG)_n_+(TCTG)_n_ and “pure” (CCTG)_n_ repetitions

The ancestral origins of the DM2 mutations containing (CCTG)_n_+(TCTG)_n_ and “pure” (CCTG)_n_ repetitions were investigated in a selected cohort of 10 families, which were chosen among our cohort of patients. Haplotype analysis was carried out using 7 STR markers within the DM2 locus (CL3N122, CL3N99, CL3N59, CL3N117, CL3N119, CL3N19, and CL3N23), and the results are presented in Supplementary Table 2. Haplotypes identified in our DM2 families were compared to the three most common DM2 European haplotypes (A, B, or C), each of which spans a core region of ∼700 kb between the CL3N122 and CL3N19 markers. Eight families are characterized by a haplotype resembling Haplotype B, while two families have a composition matching Haplotype (A) As expected, only a few samples are perfectly in accordance with the haplotypes mentioned above, in line with previously reported [22–25]. Notably, Family A, characterized by the conservation of the (TCTG)_n_ motif, presented a haplotype resembling Haplotype A, whereas in Family B, characterized by the loss of (TCTG)_n_ motif, the DM2 mutations belong to Haplotype (B) This argues in favour of a common ancestral European origin of the *CNBP* expansion in our cohort, regardless the presence/absence of the novel (TCTG)_n_ motif. It is therefore plausible to speculate that the newly described (TCTG)_n_ block may be part of the ancestral DM2 mutation, which had not been reported previously due to the technical limitations of PCR-based approaches used to characterize the *CNBP* locus prior to the development of LRS.

## Materials and methods

### DM2 patients

In this retrospective study, DNA samples were analysed from 100 genetically confirmed DM2 patients. The study was approved by the institutional review board of Policlinico Tor Vergata (Ethical Approval register numbers: 61/23), and all procedures were performed in accordance with the Declaration of Helsinki. Informed consent was obtained from all participants. All individuals were of Caucasian origin and derived from different Italian clinical centres. Samples and clinical information were anonymised immediately after collection using a unique alphanumeric identifier.

### Molecular characterisation of DM2 patients

Genomic DNA was extracted from peripheral blood using the EZ1 Advanced XL Robotic workstation (QIAGEN, Germany). Initial molecular characterisation of the DM2 mutation was performed using a combination of SR-PCR, QP-PCR and LR-PCR as described below.

### Short-range PCR

The *CNBP* locus was amplified by performing SR-PCR with the following pair of primers: CL3N58-DF (5′-GGCCTTATAACCATGCAAATG-3′) and CL3N58-DR (5′-CCTAGGGGACAAAGTGAG-3′). Amplification was carried out in a 30 µL volume, containing 50 ng of genomic DNA, PCR reaction buffer (5×), 25 mM MgCl2, 1.25 mM dNTPs, 70 pmol of each primer and 1U of Taq polymerase (Takara Bio, Mountain View, CA, USA). PCR conditions were the following: 94 °C for 3 min, 30 cycles of denaturation at 94 °C for 1 min, annealing at 58 °C for 40 s and extension at 72 °C for 40 s. A final elongation was carried out at 72 °C for 5 min. SR-PCR products were analysed by capillary electrophoresis on a 3500 Genetic Analyzer (Applied Biosystems) using GeneMapper 6 software (Applied Biosystems).

### 5′ and 3′ QP-PCR methods for the detection of “pure” (CCTG)n

80 ng of DNA were amplified in a reaction volume of 30 ul, using 1.5 units of GoTaq Polymerase (Promega), supplied buffer 1x, MgCl2 1.25 mmol/L, DMSO 10%, 1.25 mM dNTPs. Primers used for amplification of 3′end are: CL3N58-DR (Fam-5′-CCT AGG GGA CAA AGT GAG-3′) 3 pmol, P4-CCTG (5′-tac gca tcc cag ttt gag acg CCT GCC TGC CTG CCT GCC TG-3′) 0.6 pmol, P3 (5′-TAC GCA TCC CCA GTT TGA GAC G-3′) 3 pmol, and PCR conditions were the followings: 94 °C for 5 min, 30 cycles of denaturation at 94 °C for 30 s, annealing at 56 °C for 30 s and extension at 72 °C for 2 min. A final elongation was carried out at 72 °C for 10 min. For 5′ end amplification were used: DM2-Fw (Fam-5′-TTG GAC TTG GAA TGA GTG AAT G-3′) 3 pmol, P4-CAGG (5′- agc gga taa caa ttt cac aca gga CAG GCA GGC AGG CAG GCA GG-3′) 0.6 pmol, P3 (5′- AGC GGA TAA CAA TTT CAC ACA GGA-3′) 3 pmol, and PCR conditions were the followings: 94 °C for 5 min, 30 cycles of denaturation at 94 °C for 30 s, annealing at 56 °C for 30 s and extension at 72 °C for 2 min. A final elongation was carried out at 72 °C for 10 min. QP-PCR products were analysed by capillary electrophoresis on a 3500 Genetic Analyzer (Applied Biosystems) using GeneMapper 6 software (Applied Biosystems).

### LR-PCR analysis

The *CNBP* locus was amplified by performing LR-PCR with the following pair of primers: CL3N58-DF (5′-GGCCTTATAACCATGCAAATG-3′) and CL3N58-DR (5′-CCTAGGGGACAAAGTGAG-3′). Amplification was carried out in 30 µL volume, containing 9 ng of genomic DNA, PCR reaction buffer (10x), Enhancer Invitrogen Solution (10x), 50 mM MgSO_4_, DEAZA, 50pmol of each primer and 1U of Platinum Taq DNA Polymerase High Fidelity (Invitrogen, Waltham, MA, USA). PCR conditions were the following: 94 °C for 3 min, 30 cycles of denaturation at 94 °C for 1 min, annealing at 58 °C for 40 s and extension at 72 °C for 40 s. A final elongation was carried out at 72 °C for 5 min. SR-PCR products were analysed by capillary electrophoresis on a 3500 Genetic Analyser (Applied Biosystems) using GeneMapper 6 software (Applied Biosystems).

### Optimized QP-PCR method for the detection of the (TCTG)_n_ motif at the 3′ end of the (CCTG)_n_ array

**Fig. 7:**
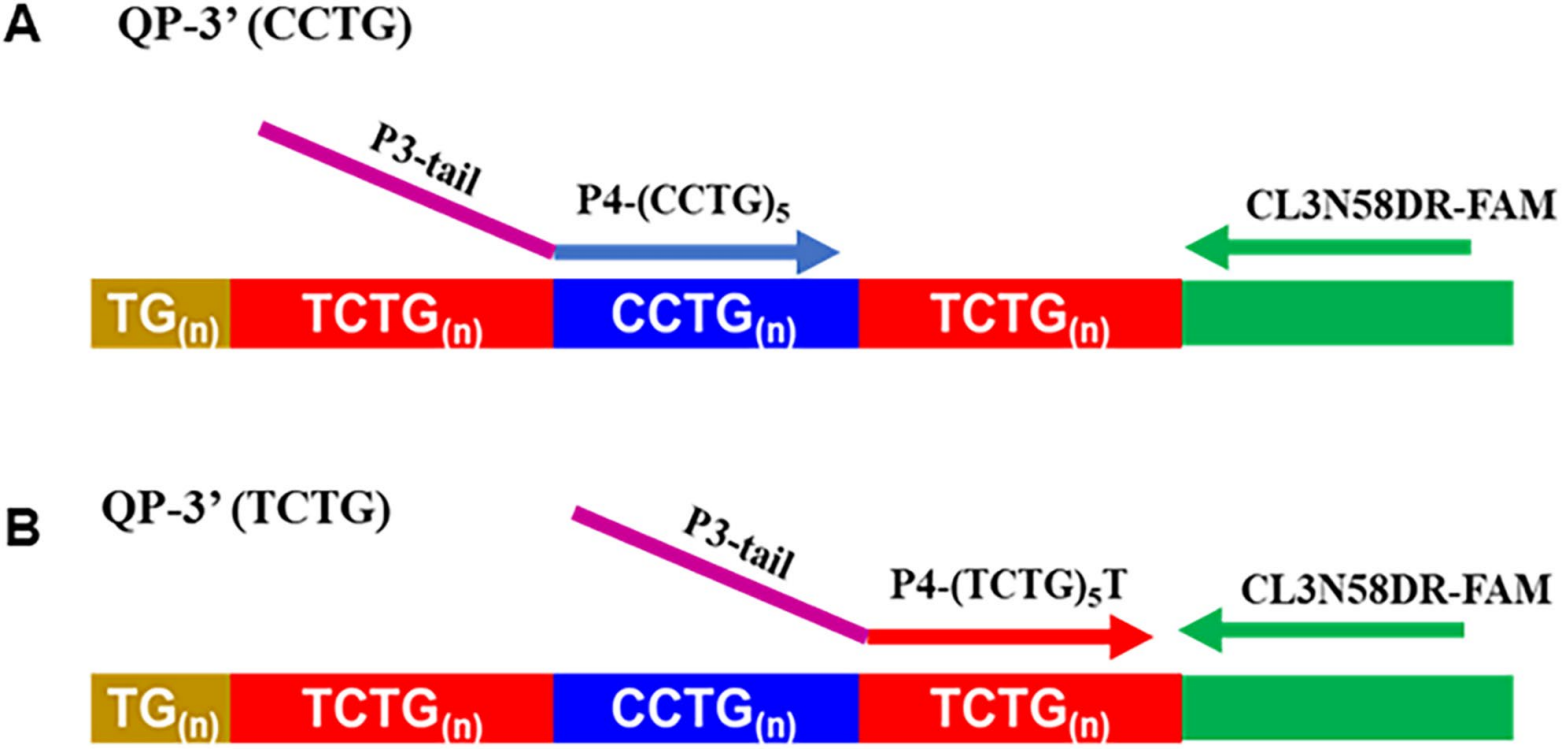
P4-TCTG primer localization for quadruplet-repeat primed PCR (QP-PCR) analysis to detect the novel (TCTG)_n_ motif. **A** P4-CCTG primer localization for standard QP-PCR at the 3′end of the (CCTG)_n_ repeated motif. **B** P4-TCTG primer localization for QP-PCR for the detection of the novel (TCTG)_n_ motif

A modified quadruplet-repeat primed PCR (QP-PCR) assay, targeting the 3′ end of the (TCTG)_n_ repeat array, was developed based on a previously published method [21]. The modified QP-PCR used the repeat primer P4-TCTG (5′-agc gga taa caa ttt cac aca ggaTCT GTC TGT CTG TCT GTC TGT**-**3′; lowercase indicates the non-complementary tail) in combination with primers CL3N58_DR-[FAM] (5′-GCC TAG GGG ACA AAG TGA GA-3′) and P3 (5′-AGC GGA TAA CAA TTT CAC ACA GGA-3′) to specifically target the 3′ (TCTG)_n_ interruptions (Fig. 7).

PCR reactions were performed in a 30 µL final volume containing 80 ng of genomic DNA, 0.3 µL GoTaq Polymerase (Promega), 2.4 µL 25 mM MgCl, 6 µL PCR Reaction Buffer,1.5 µL DMSO and 5 µL 1.25 mM dNTPs. The thermal cycling conditions were as follows: initial denaturation at 94 °C for 5 min; 30 cycles of 94 °C for 30 s, 58 °C for 30 s, and 72 °C for 4 min; and a final extension at 72 °C for 10 min. QP-PCR products were analysed by capillary electrophoresis on a 3500 Genetic Analyzer (Applied Biosystems) using GeneMapper 6 software (Applied Biosystems). To confirm the presence of the (TCTG)_n_ motif, QP-PCR products were purified using the ExoSAP-IT Express PCR Product Cleanup (Thermo Fisher Scientific) according to the manufacturer’s instructions, directly sequenced using the BigDye Terminator v3.1 Cycle Sequencing Kit (Thermo Fisher Scientific), and analysed by capillary electrophoresis on the 3500 Genetic Analyzer. Sequencing conditions were: 96 °C for 1 min, followed by 25 cycles of 96 °C for 10 s, 50 °C for 5 s, and 60 °C for 4 min. Sequencing data were analysed using Sequencing Analysis v5.2 software (Applied Biosystems).

### Southern blotting

Genomic DNA was extracted from 500 µl of anticoagulated peripheral blood using a Flexigene DNA Kit (Qiagen, Hilden, Germany). The quality and quantity of DNA were assessed using a Denovix spectrophotometer and by 1% agarose gel electrophoresis. *CNBP* expanded alleles were detected by a digoxigenin (DIG)-labeled locked nucleic acid (LNA) probe (CCTG)_5_ and analysed by our previously described protocol with some modifications [21]. Briefly, 2–3 µg of genomic DNA was digested with *AluI* and *HaeIII* restriction enzymes (New England Biolabs, Ipswich, MA). Fragments were resolved on a 0.4% agarose gel at 40 volts for 40 h. Gel was denatured, neutralised, and DNA was transferred to a positive Nylon membrane (Millipore, USA). After transfer, the membrane was fixed by cross-linking with UV-light using UV Stratalinker 2400 (Stratagene). The membrane was pre-hybridised for 30 min at 55 °C with the DIG Easy Hyb Granules solution (DIG-High Prime DNA Labelling and Detection Starter Kit II, Roche, Swiss). Hybridisation was performed overnight at 55 °C with the 10pmol/ml LNA-(CCTG)_5_ probe in the DIG Easy Hyb Granules solution (Roche, Swiss). Following hybridisation, the membrane was washed twice for 5 min in 1X SSC–0.1% SDS at room temperature, twice for 15 min in 0.5X SSC-0.1%SDS at 65 °C and once for 5 min with Washing Buffer (DIG Wash and Block buffer set, Roche, Swiss). The membrane was blocked for 30 min with Blocking Solution (DIG-High Prime DNA Labelling and Detection Starter Kit II, Roche, Swiss), followed by a 30-minute incubation with the Anti-Digoxygenin–AP antibody (1:10,000 dilution) (DIG-High Prime DNA Labelling and Detection Starter Kit II, Roche, Swiss). After washing twice, the membrane was incubated with Detection Buffer (DIG-High Prime DNA Labelling and Detection Starter Kit II, Roche, Swiss) and then with the chemiluminescent substrate CSPD Star (DIG-High Prime DNA Labelling and Detection Starter Kit II, Roche, Swiss). The membrane was then dried and developed to visualise the hybridised bands. The chemiluminescent signal was visualised on the ChemiDocTM Imaging System (Biorad, USA). The band obtained were compared with two DNA molecular weight markers, DNA Molecular Weight Marker XV (Expand™ DNA Molecular Weight Marker, Roche, Swiss) and λ DNA-*HindIII* Digest (New England Biolabs, Ipswich, MA), respectively.

### Microsatellite analysis

Haplotype analysis of the DM2 locus has been carried out by analysing seven short tandem repeats (STRs) located in the genomic region surrounding the *CNBP* gene. These markers included the centromeric markers CL3N122, CL3N99, and CL3N59 and the centromeric markers CL3N117, CL3N119, and CL3N19. The STR-based haplotypes obtained from DM2 patients were then compared to previously characterized DM2-associated haplotypes. PCRs for all markers were done with a 25 µl reaction (1.25 mM deoxynucleotide triphosphates [dNTPs], 25 mM MgCl_2_, 10X PCR Buffer II, 10µM Primer Forward and Reverse, AmpliTaq Gold™ DNA Polymerase 250U), cycled 35 times (94 °C for 45 s, 51 °C/54°C/57°C for 45 s, and 72 °C for 60 s). For each reaction, the forward (F) primer was end-labelled with FAM dye. The primer sequences employed in this study were derived from the publication by Liquori et al. [24]. PCR products were analysed by capillary electrophoresis on a 3500 Genetic Analyser (Applied Biosystems) using GeneMapper 6 software (Applied Biosystems). DM2 haplotypes were established by determining which allele co-segregated with the disease in each family. Control haplotypes were constructed by determining the alleles that were passed from parents to their offspring. If the associated allele for a marker could not be unequivocally determined, both alleles are given [23, 25, 26].

### Cas9-mediated enrichment coupled to ONT sequencing

Genomic DNA was extracted from 0.5 mL of frozen peripheral blood using the PanDNA HMW kit (PacBio, Menlo Park, CA). DNA concentration was measured using a Qubit Fluorometer with the Qubit dsDNA BR Assay Kit (Thermo Fisher Scientific, Waltham, MA). Assessment of DNA integrity was performed with the Fragment Analyzer system using the High Sensitivity Large Fragment kit (Agilent Technologies, Santa Clara, CA). Cas9-mediated enrichment of the *CNBP* repeat locus was performed as previously described [21], with the following modifications. After dephosphorylation, 2–5 µg of genomic DNA were incubated with pre-assembled ribonucleoprotein complexes (RNPs) for 60 min at 37 °C, followed by heat inactivation at 80 °C for 2 min. ONT sequencing adapters (LA) from the SQK-LSK114 kit (ONT, Oxford, UK) were ligated to the cleaved, dA-tailed target ends for 60 min at room temperature. The reaction was stopped by adding one volume of 10 mM Tris-EDTA (pH 8.0). Short fragments (< 3 kb) and residual enzymes were removed using 0.34× AMPure XP beads (Beckman Coulter, Brea, CA), followed by two washes with Long Fragment Buffer (ONT). The library was eluted by incubating the beads in ONT elution buffer (EB) for 30 min at room temperature, mixed with 37.5 µL of Sequencing Buffer and 25.5 µL of Library Loading Beads (ONT) and then loaded onto a primed FLO-MIN114 (R10.4.1) flow cell. Sequencing was performed on a MinION device using the MinKNOW software (ONT), until the sequencing yield reached a plateau.

Up to 3 samples were sequenced for each flow-cell, washing with EXP-WSH004kit (ONT) between each other. Raw pod5 files were basecalled using Dorado v0.8.3 with the Super Accurate model (SUP) model 4.3.0, with parameters “basecaller -x cuda:0 --min-qscore 10 -r sup@v4.3.0 $POD5_DIR. Only pass reads with quality score of 10 were used for the subsequent analysis. Basecalled reads were aligned to the human reference genome (hg38) using Minimap2 (v2.28) using the -ax map-ont preset to tailor the mapping algorithm for ONT data. Secondary alignments were excluded by setting the --secondary = no flag to retain only primary mappings. The resulting alignments were output in SAM format for downstream analysis. Reads spanning the entire expanded repeat region (“complete reads”) were extracted as previously described [21] and analyzed using MosaicViewer (https://github.com/MaestSi/MosaicViewer_*CNBP*) with the parameters: -minlength 16 and -minratio 75. Annotated repeat motifs were visualized in the Integrative Genome Visualization tool [27] and graphical representations were generated with R packages (“ggplot2”).

### Statistical analyses

The data were analyzed using GraphPad Prism version 10. Unpaired Student’s t-test and a Spearman Correlation analysis were used to assess group differences. Values shown in figures represent the means ± standard deviation (SD). Statistical significance was established at *p < 0.05 and ** *p* < 0.01.

## Discussion

This study further confirms and extends our previously published data on the molecular characterisation of DM2 patients using LRS and PCR-based techniques [21]. In the paper from Alfano et al. [21], Cas9-targeted ONT LRS allowed the discovery, for the first time, of a novel (TCTG)_n_ motif downstream of the (CCTG)_n_ array in 7 of 9 DM2 patients [21]. A modified QP-PCR assay, targeting the 3′ end of the (TCTG)_n_ repeat array, was also developed, relying on the use of the allele-specific repeat primer P4-TCTG. Analysis of a larger cohort of genetically confirmed Italian DM2 patients (*n* = 100) revealed that this additional non-CCTG repeat is present in the majority of *CNBP* expansions (88%), consistent with the findings of Wendlandt et al. [28] and represents a general feature of the DM2 mutation. In contrast, variant repeat-containing alleles (VRs), containing non-CTG repeats (CCG, GGC, CTC, CAG), are rare in DM1 patients [28, 29] (less than 3–11% of patients), where they act as *in cis* modifiers of the clinical phenotype also stabilizing repeat number across generations.

In addition, non-CCTG sequences at the DM2 locus are confined to the 3′ region and not interspersed within the (CCTG)_n_ tract. This polarized localization may have different effects on the dynamics and trans-acting mechanisms of the DM2 compared to the DM1 expansions. Our optimized QP-PCR assay also allows us to re-evaluate DM2 patients with previous partial negative results using the standard QP-PCR assay at the 3′end of the CCTG array, increasing the sensitivity of the DM2 genetic test. In accordance with the International Best Practise Guidelines and Recommendation, molecular testing for DM2 typically involves a first short-range PCR step and, if only one *CNBP* allele is detected, subsequent techniques such as QP-PCR and or Southern blotting of genomic DNA or long-range PCR products are used to detect (CCTG)_n_ repeat expansions [12]. It is worth noting that the detection of the DM2 expansion, in either QP-PCR or LR-PCR, relies on the use of repeated primers and probes complementary to the “pure” (CCTG)_n_ array. The presence of non-CCTG repetitions can therefore lead to false negatives in standard PCR-based approaches, underscoring the need to refine the molecular diagnostic workflow to enhance the detection rate. Based on our and other previous results [21, 28, 30], we strongly recommend performing bidirectional QP-PCR in the routine DM2 diagnostic workflow, since downstream non (TCTG)_n_ repeat may cause false negative results if only standard 3′ unidirectional QP-PCR is used. Based on the data available so far, QP-PCR at the 5′ and 3′ ends of the (CCTG)_n_ array can detect the majority of the DM2 expanded alleles and can be easily adopted by standard laboratories using the unidirectional downstream QP-PCR since it does not require additional expertise or equipment. This approach, already in use in our laboratory, identified 9 false-negative patients with standard 3′ QP-PCR out of a cohort of 100 patients with clinical suspicion of DM2, giving QP-PCR alone, at the 3′ end, a sensitivity of 91%. When the QP-PCR signal at the 3′ end is unclear, with a low-intensity repeat stutter pattern or yields controversial results compared to the upstream end (negative vs. positive), the potential presence of an additional (TCTG)_n_ repeat or a more complex repeat pattern may be suspected. At this point, to detect the presence of a (TCTG)_n_-containing expansion within the *CNBP* gene, we propose to adopt our optimized 3′ QP-PCR protocol with the use of P4-TCTG primer.

The modified QP-PCR assay validated in this study enhances the molecular diagnosis workflow for DM2, decreasing the risk of false-negative results at the 3′ end of the (CCTG)_n_ array.

**Fig. 8:**
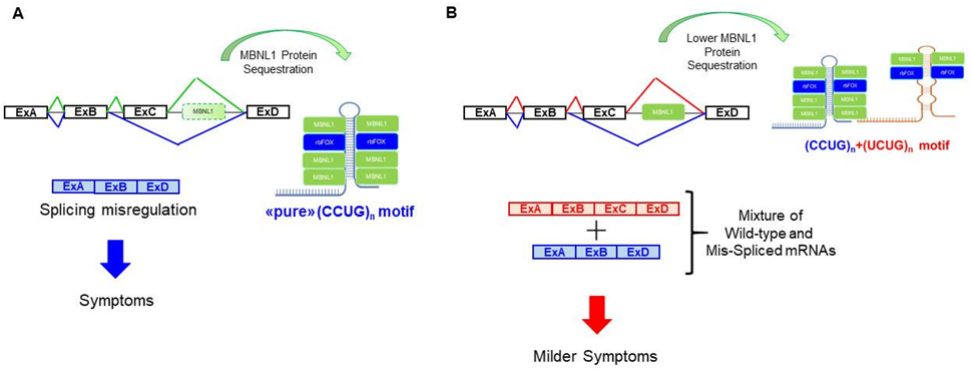
Hypothetical pattern of splicing alteration in DM2. **A** Classical mechanism of splicing alteration in DM2 patients with “pure” (CCTG)_n_ motif. This leads to severe splicing mis-regulation (e.g., exclusion of exon C) and typical DM2 clinical symptoms. **B** Hypothetical mechanism of splicing alteration in DM2 patients with the (CCTG)_n_+(TCTG)_n_ motif. The presence of (TCTG)_n_ interruptions is hypothesised to reduce the efficiency of MBNL1 protein sequestration. However, as some MBNL1 still binds to the (CCUG)_n_ portion, a mixture of wild-type and mis-spliced mRNA transcripts is produced. This may result in a milder splicing mis-regulation and, consequently, a milder clinical phenotype

The observed complexity of *CNBP* repeat expansions, along with pronounced variability in expansion size and somatic instability, hampers the establishment of definitive genotype–phenotype correlations. In addition to the technical difficulties in identifying non-(CCTG)_n_ repeat motifs, their presence may contribute, at least in part, to modulation of the clinical phenotype in DM2 patients. In line with this hypothesis, our preliminary genotype–phenotype correlation data suggest a possible effect of “pure” (CCTG)_n_ *vs* (CCTG)_n_+(TCTG)_n_ alleles on the age of disease onset, which appears to be earlier when the (TCTG)_n_ tract is not present. On the other hand, no significant differences in the clinical symptoms have been observed between the two groups, suggesting that the sole presence or absence of the (TCTG)_n_ motif does not adequately stratify the clinical phenotype accordingly to the severity of the reported symptoms. It is still necessary to point out that our DM2 patients come from different Italian neuromuscular centres, each employing not very well standardized clinical criteria. Moreover, the limited number of patients without the (TCTG)_n_ motif (n = 12), considerably reduces the significance of these observations that need to be confirmed in larger cohorts of patients. Traditional methods cannot resolve the DM2 expansion to the single-nucleotide level, and they have a low ability to detect larger alleles and the degree of somatic mosaicism, which are important elements to consider in genotype–phenotype correlation studies. This limitation can be overcome with LRS, an amplification-free analysis that simultaneously quantifies and characterizes the entire *CNBP* expanded locus, as described in our work and previous studies [21, 31]. The DM2 locus has been fully characterised at the single-nucleotide level in 9 DM2 patients, confirming the presence/absence of the (TCTG)_n_ motif revealed by QP-PCR and the usefulness of this method within the DM2 diagnostic workflow. Our analysis underscored the considerable potential of LRS in determining both the composition and the degree of somatic mosaicism of the DM2 expansion. LRS analysis still leaves some open questions, as certain regions could not be classified into either of the two motifs. This phenomenon was more pronounced in some patients: for example, results from Family B were relatively clear, whereas those from Family A showed a higher proportion of unclassified read portions. Although we cannot fully exclude subtle experimental variation, the qualitative variations in repeat motif resolution observed between family A and family B were not associated with any identifiable technical factor or differences in sequencing data quality. We would exclude that this result is specifically linked to Family A as similar results were observed also for an unrelated patient (DM2-13). If these variations reflect intrinsic run-to-run variability of the ONT platform, they may influence data interpretation and should be acknowledged as a potential limitation of the technology in the context of DM2 repeat analysis. At least part of the repeat stretches that could not be classified as either CCTG or TCTG (visualized as grey regions) appear to be linked to ONT sequencing errors. Inspection of the raw reads suggests that these segments often correspond to TCTG repeat stretches interrupted by sequencing errors (Supplementary Fig. 3). It is well established that ONT sequencing is prone to higher error rates in homopolymeric and highly repetitive regions. Repeat variations artefacts have been reported in other repeat expansion contexts, including DM1 [32], and variability linked to the basecallers have also been documented in relation to repeat analysis [33]. In light of this evidence, we are confident that the grey stretches are at least partially attributable to ONT sequencing and basecalling limitations. To minimize the impact of such errors and to obtain a reliable and biologically meaningful representation of repeat structure, we applied a threshold of four contiguous repeat units (i.e., ≥ 16 bp) to define a given motif. Sequences shorter than this threshold were therefore not classified, which likely accounts, at least in part, for the unclassified repeat stretches observed in some patients. Nevertheless, the presence of additional repeat motifs or atypical/not-reported interruptions cannot be excluded and could determine to the inability to confidently classify certain read segments as either CCTG or TCTG. A definitive resolution of these issues would require dedicated orthogonal validation, ideally using PacBio sequencing in combination with the Cas9 amplification-free enrichment strategy, which is now commercially available (PureTarget kit). Future studies should therefore directly compare ONT- and PacBio-based LRS analyses of DM2 repeat expansions in parallel on the same patient samples. Overall, our data demonstrated that the structure of the *CNBP* expanded allele is far more complex than previously understood. Such complexity underscores the significant challenges involved in accurately stratifying DM2 patients for reliable genotype–phenotype correlations. Beyond characterizing the detailed structure of *CNBP* repeat expansions, LRS facilitates the evaluation of somatic mosaicism, which represents a critical parameter to consider in this context. Our analyses revealed that the fraction of sequencing reads from the expanded allele carrying the (TCTG)_n_ motif varied between 35.5% and 88.8%, reflecting high degree of variability across samples. We would exclude that this variability is attributable to limitations of ONT sequencing. Even in the “cleanest” ONT runs, we consistently observed reads containing only the canonical (CCTG)_n_ motif, with just a subset of reads carrying the (TCTG)_n_ motif. Importantly, the median coverage achieved for the expanded alleles was 84× and exceeded 50× in all patients. This level of coverage provides sufficient sensitivity to reliably detect low-frequency events down to approximately 2% (i.e., 1 in 50 cells). In addition, a variable fraction of molecules carrying the TCTG motif was observed not only in the present study but also in our previous work [21], which was performed using a different sequencing chemistry, as well as in an independent report from another group [28]. It is therefore possible to speculate that the effect of the DM2 expansion on the phenotype derives from a complex interaction between the length, composition, and level of somatic mosaicism in a single patient. Future studies utilising LRS and other orthogonal methods will be essential for a more thorough characterisation of the motifs within the *CNBP* expanded allele and to include these technologies in the routine molecular diagnostic workflow of medical genetics laboratories. We were also able to analyse the dynamics of the (TCTG)_n_ motif transmission in 16 DM2 families using the modified QP-PCR. Our results consistently showed that the (TCTG)_n_ array was inherited without any occurrences of *de novo* mutations. The study of the intergenerational transmission of the DM2 mutation in two families (3 meiotic transmissions) using LRS indicates that the (TCTG)_n_ motif may be conserved or lost when the DM2 expanded alleles contracted as in Family B. Overall, we confirm the instability of the DM2 mutation together with its tendency to contract rather than expand across generations as reported in the paper by Day et al. [34]. The main limitation of this analysis is the variability in the age at sampling of each patient which can influence the repeat size. As already reported in DM1, the gold standard to study the intergenerational dynamics of the repeat avoiding age at sampling-bias, is the determination of estimation the progenitor allele length (ePAL) [35]. Unfortunately, there is not enough data allowing the determination of this important genetic parameter based on LRS data in DM2. Additional studies comparing Small-Pool PCR (SP-PCR), with LRS results are necessary to solve this issue, until then. For this reason, we decided to utilize the maximum, minimum and median repeat length to analysed the intergenerational dynamics of the DM2 expansion in our two families. Haplotype analysis of the DM2 locus is consistent with a common ancestral European origin of the DM2 mutation in our cohort, regardless of the presence/absence of the novel (TCTG)_n_ motif. It is therefore plausible to hypothesize that this recently described (TCTG)_n_ block represents an intrinsic component of the original DM2 mutation, which may have remained undetected in previous studies due to technical limitations of PCR-based methodologies. Many complex mechanisms, likely involving the formation of alternative DNA structures, are responsible for repeat instability observed in repeat expansion disorders, including DM2 [36, 37]. (CCTG)_n_ repeated regions can form hairpins containing mismatches, called imperfect hairpins, which escapes faithful DNA replication, transcription or repair [36] leading to large-scale expansion/contractions. The secondary structures and high GC content of the expanded (CCTG)_n_ repeats tend to facilitate RNA-DNA hybrid (R-loop) formation during transcription [38] which are highly susceptible to damage [39, 40]. Moreover, multiple slippage events can arise as the DNA polymerase copies the repeated tract, where the DNA strands temporarily denature and misalign, causing the (CCTG)_n_ repeat to expand or contract. The detailed mechanisms of instability depend on the repeat length and nucleotide sequences, as well as the cell type examined. Variations in the composition of repeated motifs of the DM2 mutation may therefore affect the level of somatic and germinal instability. In general, the total length of a “pure” (CCTG)_n_ tract rather than the repeat length overall predicts the level of repeat stability. In DM1, for instance, the presence of non-(CTG)_n_ interruptions stabilizes the (CTG)_n_ expansion even through maternal transmission. So far, non-(CCTG)_n_ repetitions have been reported only in the 3′end of the (CCTG)_n_ array, adjacent to a (TCTG)_4_ tract localized downstream of the (CCTG)_n_ in wild-type *CNBP* alleles [21, 31]. This polarization may suggest a mechanism of instability involving DNA repair pathway mediated by incorrect recombination events [41]. In this context, it is important to emphasize that additional (TCTG)_n_ motifs are contained in the (TG)_v_(TCTG)_w_ repeated region 5′ to the (CCTG)_n_ array both in wild-type and expanded *CNBP* alleles. In addition, we must also consider that the presence of non-(CCTG)_n_ repeats may modulate the *in trans*, RNA gain-of-function, mechanism of the expanded *CNBP* transcripts. Both MBNL1 and rbFOX1 compete for binding to expanded (CCUG)_n_ repeats, with overexpression of rbFOX1 reducing MBNL1’s sequestration in RNA foci and improving splicing defects and muscle atrophy in DM2 models [42]. This competition may explain some of the phenotypic differences between DM1 and DM2, as rbFOX1 binds to (CCUG)_n_ repeats but not the (CUG)_n_ repeats associated with DM1. Differential interaction and affinity with RNA-binding proteins of “pure” (CCTG)_n_ and (TCTG)_n_-containing *CNBP* alleles may have different effects on the pathogenic mechanism at the basis of DM2, partially explaining the variable expressivity of the DM2 phenotype (Fig. 8).

Exploring the complex RNA-protein interaction of the *CNBP* expanded transcripts with different nucleotide composition demands a comprehensive suite of future studies, which fall outside the scope of this work.

In conclusion, long-read sequencing technology is now revolutionizing the study and detection of repeat expansion disorders. Deciphering the complete molecular characterisation of the *CNBP* locus achieved through LRS represents a significant advancement also in the genetic analysis of DM2. By enabling the detailed identification of repeat structure and sequence composition, particularly of the (CCTG)_n_ expansion and different motifs, this approach enhances the overall sensitivity and accuracy of DM2 genetic testing, providing new opportunities for understanding the molecular basis of disease variability and more refined genotype–phenotype correlations. Moreover, such high-resolution genotyping has the potential to refine patient stratification, which is crucial for future personalized therapeutic treatments.

## Supplementary Information

Supplementary Material 1.

Supplementary Material 2.

## Data Availability

The datasets used and/or analysed during the current study are available from the corresponding author on reasonable request.
