# Supplementary figures and images for: The novel (TCTG)n motif in CNBP expanded alleles: composition, dynamics and genotype–phenotype correlation in Myotonic dystrophy type 2 (DM2)
Source: Hum Genomics. 2026 Apr 5;20:87. doi: 10.1186/s40246-026-00954-7 (PMC13195993; doi:10.1186/s40246-026-00954-7)

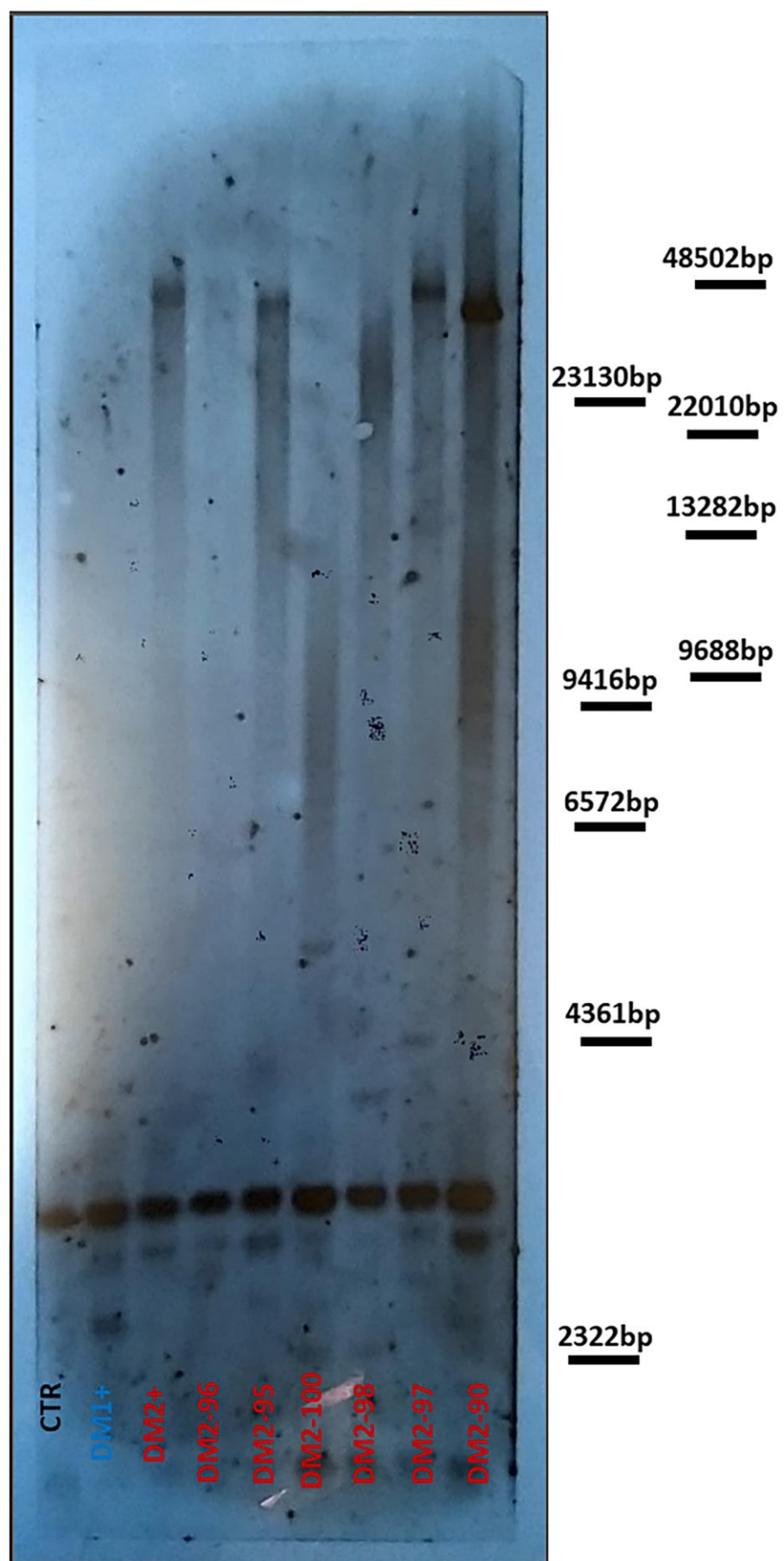

Supplement: Supplementary file 1 — Supplementary Material 1. [file 40246_2026_954_MOESM1_ESM.pdf]
