# Supplementary material for: The novel (TCTG)n motif in CNBP expanded alleles: composition, dynamics and genotype–phenotype correlation in Myotonic dystrophy type 2 (DM2)
Source: Hum Genomics. 2026 Apr 5;20:87. doi: 10.1186/s40246-026-00954-7 (PMC13195993; doi:10.1186/s40246-026-00954-7)
Supplement: Supplementary file 2 — Supplementary Material 2. [file 40246_2026_954_MOESM2_ESM.docx]

**Supplementary Materials**

**
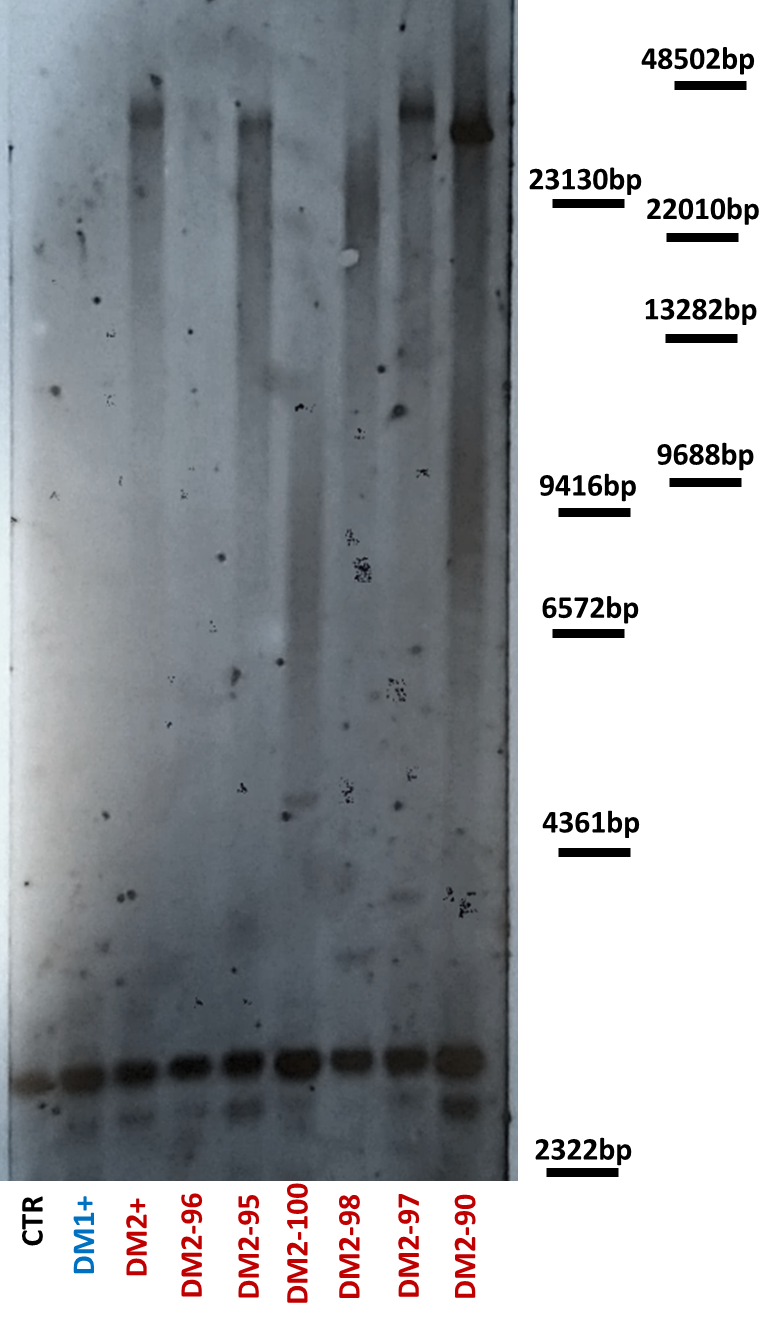
**

**Figure Supplement 1 Southern blot Analysis of DM2 patients with “pure” (CCTG)_n_ repeats.** Southern blot analysis of genomic DNA double-digested and probed with (LNA)-labelled (CCTG)_5_ locked nucleic acid probe. Lane 1, CTR, healthy control sample; lane 2, DM1 sample; lane 3, DM2 sample as a positive control and lanes 4–9, DM2 samples with “pure” (CCTG)_n_ motif. Molecular markers are indicated on the right.

**Supplement Table 1: *CNBP* expanded allele length and age at onset in DM2 patients with “pure” (CCTG)_n_ repeated motif.**

| DM2 patient | Repeat Length (bp) | Age at onset |
| --- | --- | --- |
| DM2-90 | 35,000bp | 33 |
| DM2-95 | 40,000bp | Childhood |
| DM2-96 | 40,000bp | 45 |
| DM2-97 | 40,000bp | 39 |
| DM2-98 | 23,000bp | 18 |
| DM2-100 | 9,400bp-6,500bp | 30 |


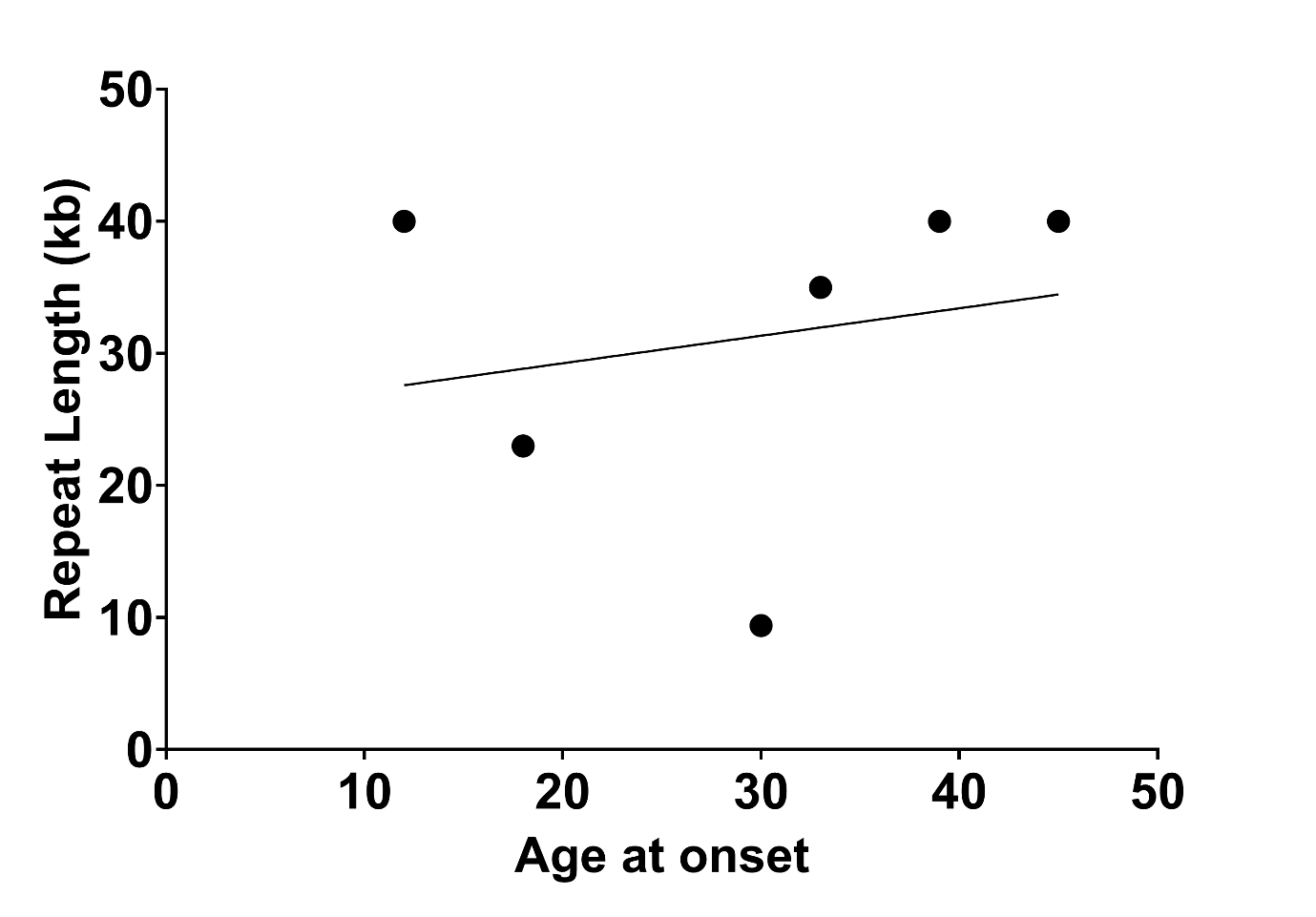


**Figure Supplement 2. Correlation Analysis** **in DM2 Patients Carrying the “pure” (CCTG)_n_ Motif. Spearman correlation analysis between the maximum length of the upper edge of the Southern blot trace for the expanded allele and the age of onset in “pure” (CCTG)_n_ DM2 patients (n = 6) (r=0.3339; *p*=0.5333)**

**Supplement Table 2: Haplotype of 10 DM2 Families**

| **FAMILIES** |  | ***CL3N122*** | ***CL3N99*** | ***CL3N59*** | ***DM2 Exp*** | ***CL3N117*** | ***CL3N119*** | ***CL3N19*** | ***CL3N23*** |
| --- | --- | --- | --- | --- | --- | --- | --- | --- | --- |
|  | **HAPLOTYPE B** | **216** | **181** | **157/148** |  | **241/245** | **240** | **190** | **238** |
|  | (Liquori *el al*.,) |  |  |  |  |  |  |  |  |
| **DM2-B** | DM2-B1 | 214/216 | 181 | 157 |  | 241 | 242/240 | 190 | 250 |
|  | DM2-B2 | 214/216 | 181 | 157 |  | 241 | 242/240 | 190 | 250 |
| **DM2-C** | DM2-C1 | 216 | 175 | 147 |  | 241/245 | 234 | 190 | 238 |
|  | DM2-C2 | 216 | 175 | 147 |  | 241/245 | 234 | 190 | 238 |
|  | DM2-C3 | 216 | 175 | 147 |  | 241/245 | 234 | 190 | 238 |
| **DM2-D** | DM2-D1 | 214 | 181 | 157/148 |  | 241/245 | 234 | 190 | 242 |
|  | DM2-D2 | 214 | 181 | 157/148 |  | 241/245 | 234 | 190 | 242 |
| **DM2-E** | DM2-E1 | 216 | 191 | 157 |  | 241 | 242 | 190 | 234 |
|  | DM2-E2 | 216 | 191 | 157 |  | 241 | 242 | 190 | 234 |
| **DM2-G** | DM2-G1 | 214 | 183 | 147/141 |  | 241 | 240/238 | 190 | 242 |
|  | DM2-G2 | 214 | 183 | 147/141 |  | 241 | 240/238 | 190 | 242 |
| **DM2-H** | DM2-H1 | 210 | 205 | 145 |  | 241/245 | 240 | 180 | 242 |
|  | DM2-H2 | 210 | 205 | 145 |  | 241/245 | 240 | 180 | 242 |
| **DM2-I** | DM2-I1 | 210 | 175 | 147 |  | 241/245 | 240 | 180 | 242 |
|  | DM2-I2 | 210 | 175 | 147 |  | 241/245 | 240 | 180 | 242 |
| **DM2-L** | DM2-L1 | 214 | 191 | 157 |  | 241/245 | 236 | 198 | 242 |
|  | DM2-L2 | 214 | 191 | 157 |  | 241/245 | 236 | 198 | 242 |
|  | DM2-L3 | 214 | 191 | 157 |  | 241/245 | 236 | 198 | 242 |
|  | **HAPLOTYPE A** | **216** | **185** | **157** |  | **241** | **236/232** | **208** | **234/230** |
|  | (Liquori *el al.,*) |  |  |  |  |  |  |  |  |
| **DM2-A** | DM2-A5 | 216 | 185 | 157/148 |  | 241/245 | 240 | 208 | 242 |
|  | DM2-A7 | 216 | 185 | 157/148 |  | 241/245 | 240 | 208 | 242 |
|  | DM2-A6 | 216 | 185 | 157/148 |  | 241/245 | 240 | 208 | 242 |
| **DM2-F** | DM2-F1 | 214/216 | 185 | 157 |  | 241/245 | 240/238 | 210 | 238 |
|  | DM2-F2 | 214/216 | 185 | 157 |  | 241/245 | 240/238 | 210 | 238 |
|  | **HAPLOTYPE C** | **218** | **175** | **150** |  | **241** | **240** | **190** | **226/228** |
|  | (Liquori *et al.,*) |  |  |  |  |  |  |  |  |

**Haplotypes composed by STR markers already described by Liquori et al., shown in the first row. Marker lengths from the original work have been recalculated following the work by Damen et al. Patients have been assigned to a haplotype based on the repeated lengths of the disease-linked alleles. Eight families (namely DM2-B, DM2-C, DM2-D, DM2-E, DM2-G, DM2-F, DM2-H and DM2-L) presented consistent results, confirming B is the most frequent haplotype. Minor differences in the consensus are represented with a lighter colour. Two families (DM2-A, DM2-F) are in consensus haplotype A. No consensus haplotype C was found.**


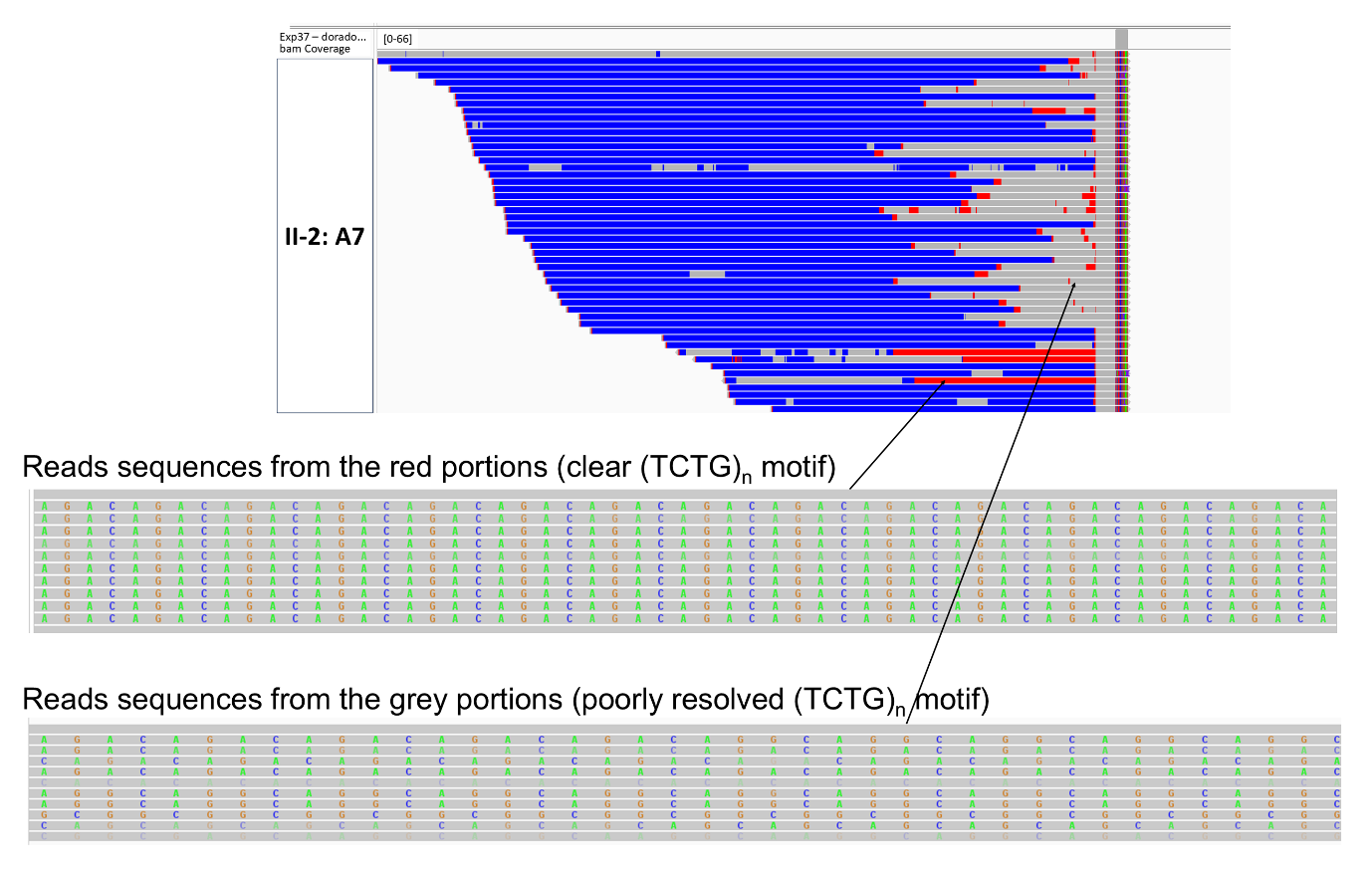


**Figure Supplement 3.** Integrative Genomics Viewer (IGV) visualisation (18 kb window) of ONT targeted sequencing data showing complete reads from the expanded allele of patient A7, aligned at the 3′ end. The (CCTG)n repeat motif is shown in blue, the (TCTG)_n_ motif in red, and unresolved repeat segments are highlighted in grey. Magnified views in the lower panels illustrate examples of reads carrying clearly resolved (TCTG)n motif, as well as sequences within the grey regions. The latter sequences show (TCTG)_n_ repeat tracts interspersed with apparent random errors, leading to a red–grey-red pattern.


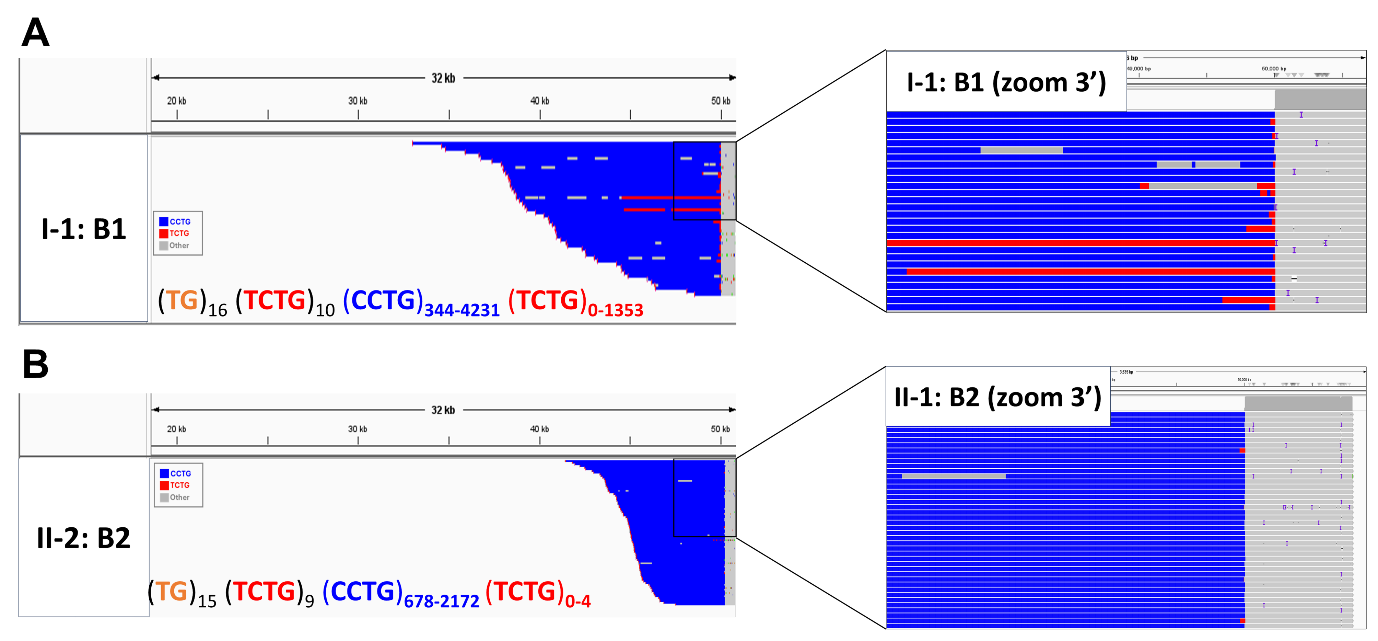


**Figure Supplement 4.** Integrative Genomics Viewer (IGV) visualisation (32kbp windows) of ONT-targeted sequencing data of patient B1 (A) and patient B2 (B). Left panels report the same data shown in Figure 6C, showing complete reads derived from the expanded alleles aligned at the 3′ end. Right panels show a higher-magnification views of the same alignments, highlighting reads including the (TCTG)n motif (red) at 3’ end and reads characterized by ‘pure’ (CCTG)n expansion (blue).
